# Vaccination with Glycan-Modified HIV NFL Envelope Trimer-Liposomes Elicits Broadly Neutralizing Antibodies to Multiple Sites of Vulnerability

**DOI:** 10.1016/j.immuni.2019.10.008

**Published:** 2019-11-19

**Authors:** Viktoriya Dubrovskaya, Karen Tran, Gabriel Ozorowski, Javier Guenaga, Richard Wilson, Shridhar Bale, Christopher A. Cottrell, Hannah L. Turner, Gemma Seabright, Sijy O’Dell, Jonathan L. Torres, Lifei Yang, Yu Feng, Daniel P. Leaman, Néstor Vázquez Bernat, Tyler Liban, Mark Louder, Krisha McKee, Robert T. Bailer, Arlette Movsesyan, Nicole A. Doria-Rose, Marie Pancera, Gunilla B. Karlsson Hedestam, Michael B. Zwick, Max Crispin, John R. Mascola, Andrew B. Ward, Richard T. Wyatt

**Affiliations:** 1 Department of Immunology and Microbiology, The Scripps Research Institute, La Jolla, CA 92037, USA; 2 International AIDS Vaccine Initiative, Neutralizing Antibody Center at The Scripps Research Institute, La Jolla, CA 92037, USA; 3 Department of Integrative Structural and Computational Biology, The Scripps Research Institute, La Jolla, CA 92037, USA; 4 School of Biological Sciences, University of Southampton, Southampton, UK; 5 Vaccine Research Center, National Institute of Allergy and Infectious Diseases, NIH, Bethesda, MD 20892, USA; 6 Department of Microbiology, Tumor and Cell Biology, Karolinska Institutet, Stockholm 171 77, Sweden; 7 Vaccine and Infectious Disease Division, Fred Hutchinson Cancer Research Center, Seattle, WA 98109, USA; 8 Center for HIV/AIDS Vaccine Immunology and Immunogen Discovery, The Scripps Research Institute, La Jolla, CA 92037, USA

**Keywords:** HIV-1, Env, NFL, trimer, liposomes, 1C2, E70, glycan deletion, vaccine, bNAbs

## Abstract

The elicitation of broadly neutralizing antibodies (bNAbs) against the HIV-1 envelope glycoprotein (Env) trimer remains a major vaccine challenge. Most cross-conserved protein determinants are occluded by self-N-glycan shielding, limiting B cell recognition of the underlying polypeptide surface. The exceptions to the contiguous glycan shield include the conserved receptor CD4 binding site (CD4bs) and glycoprotein (gp)41 elements proximal to the furin cleavage site. Accordingly, we performed heterologous trimer-liposome prime:boosting in rabbits to drive B cells specific for cross-conserved sites. To preferentially expose the CD4bs to B cells, we eliminated proximal N-glycans while maintaining the native-like state of the cleavage-independent NFL trimers, followed by gradual N-glycan restoration coupled with heterologous boosting. This approach successfully elicited CD4bs-directed, cross-neutralizing Abs, including one targeting a unique glycan-protein epitope and a bNAb (87% breadth) directed to the gp120:gp41 interface, both resolved by high-resolution cryoelectron microscopy. This study provides proof-of-principle immunogenicity toward eliciting bNAbs by vaccination.

## Highlights

• Removal of N-glycans proximal to the CD4 binding site increases B cell accessibility
• Heterologous Env trimer-liposome regimen drives B cells to cross-conserved sites
• Vaccine elicitation of an N-glycan-dependent CD4 binding site neutralizing antibody
• Elicitation of an interface-directed antibody with 87% HIV neutralization breadth

## Graphical Abstract

**Figure:**
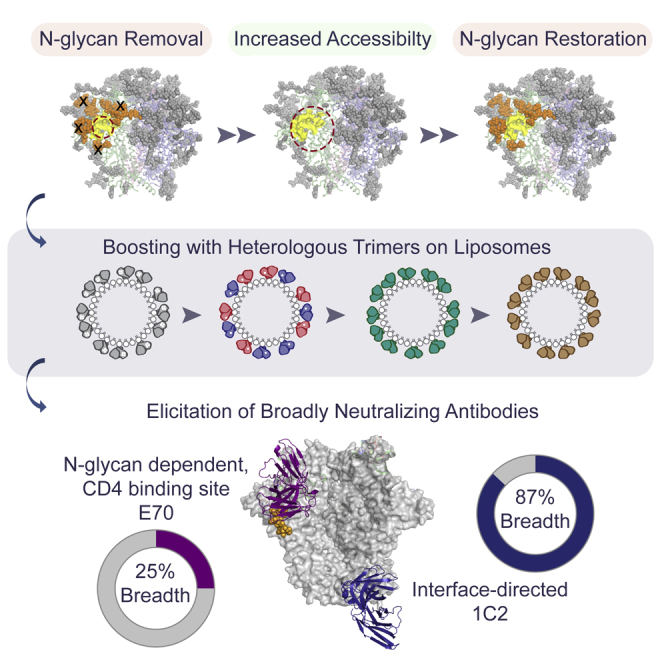

## Teaser

Eliciting broadly neutralizing HIV antibodies by vaccination remains a challenge. Dubrovskaya et al. use an immunization regimen incorporating targeted N-glycan removal and heterologous prime:boosting with NFL trimer-liposomes in rabbits to elicit broadly neutralizing responses to cross-conserved HIV-1 epitopes, including an antibody with 87% neutralization breadth. Further structural analyses highlight similarities between the vaccine-elicited antibodies and the human broadly neutralizing antibodies.

## Introduction

The HIV-1 surface-exposed functional Env spike is the sole virally encoded target for broadly neutralizing antibodies (bNAbs). Remarkably, such bNAbs can neutralize diverse clinical strains but arise sporadically during the course of natural infection and usually after 2 or more years (McCoy and Burton, 2017). To elicit such bNAbs by vaccination, a major effort is to design soluble mimetics that faithfully recapitulate structural features of the viral spike. The development of the well-ordered, soluble BG505 SOSIP.664 Env trimer ushered in a new era of HIV-1 spike mimetics (for review, see Sanders and Moore, 2017). The well-ordered SOSIP trimer adopts a near-native conformation, as confirmed by high-resolution structural analysis (Julien et al., 2013, Lyumkis et al., 2013, Pancera et al., 2014). Since this initial discovery, multiple efforts to produce more stable or homogeneous, soluble Env mimetics derived from various HIV-1 strains have been pursued. These include the cleavage-independent, native flexibly linked (NFL) trimers that we developed (Guenaga et al., 2015a, Guenaga et al., 2017, Karlsson Hedestam et al., 2017, Sharma et al., 2015), uncleaved prefusion-optimized trimers (Kong et al., 2016), and modified SOSIPs (Sanders and Moore, 2017). While these well-ordered trimers are efficiently recognized by HIV-directed bNAbs, their use in prime:boost strategies in animal models mostly elicit autologous NAbs to strain-restricted epitopes and not to cross-conserved elements needed for a broadly effective vaccine (Feng et al., 2016, Klasse et al., 2018, Martinez-Murillo et al., 2017, Pauthner et al., 2017, Sanders and Moore, 2017, Sanders et al., 2015, Torrents de la Peña et al., 2017, Torrents de la Peña and Sanders, 2018).

Most cross-conserved sites on the Env spike are occluded by host-derived N-glycans that appear as “self” and sterically limit naive B cell recognition of the underlying polypeptide surface. Functional gaps in the contiguous glycan shield are the protein surfaces of the receptor CD4 binding site (CD4bs) and conserved residues proximal to the furin cleavage site at the Env interface. Besides these sites of Ab vulnerability, bNAbs directed to the V2 apex, the N332 glycan supersite and to the high-mannose patch are elicited in some patients during chronic HIV-1 infection (McCoy and Burton, 2017). However, rarely have such bNAbs been elicited by Env vaccination in outbred subjects. One exception is the elicitation of broadly neutralizing activity following peptide-trimer prime:boosting that is directed to the Env fusion peptide (FP) (Xu et al., 2018). This peptide determinant is centrally located in a well-conserved gp41 region that is partially devoid of N-glycans, allowing accessibility for furin-mediated cleavage and liberation of the FP for viral entry.

To elicit vaccine-induced bNAbs, we integrated heterologous Env trimer prime:boosting with liposomal multi-valent particulate array to drive B cell responses directed at cross-conserved sites. We preferentially exposed the CD4bs by eliminating proximal N-glycans to this region, while maintaining the native-like state of the cleavage-independent NFL trimers. As we have shown previously, targeted glycan deletion efficiently activates a myriad of polyclonal B cells directed to the CD4bs. We reasoned that subsequent immunizations with the N-glycans restored, coupled with heterologous Env trimers, could then boost and selectively drive B cells still capable of accessing the CD4bs to become broadly neutralizing (Dubrovskaya et al., 2017). We demonstrated previously that high-density trimer array on synthetic liposomes enhances B cell activation and the elicitation of NAbs (Bale et al., 2017, Ingale et al., 2016). Here, we show that this multi-faceted approach elicited cross-neutralizing serum immunoglobulin G (IgG) Abs in a subset of rabbits and that much of the response was directed to the CD4bs. We isolated cross-neutralizing Abs and demonstrated that one Ab was directed to the CD4bs and the other to the gp120:gp41 interface region, as confirmed by serological mapping and high-resolution electron microscopy (EM). We conclude that glycan deletion on native-like trimers and heterologous boosting, coupled with particulate array, is an effective means to elicit Env-specific Abs capable of cross-neutralizing multiple difficult-to-neutralize (tier 2) HIV-1 clinical isolates.

## Results

### Glycan Modification of Heterologous NFL Trimers Preferentially Exposes the CD4bs while Maintaining Trimer Integrity

**Figure 1:**
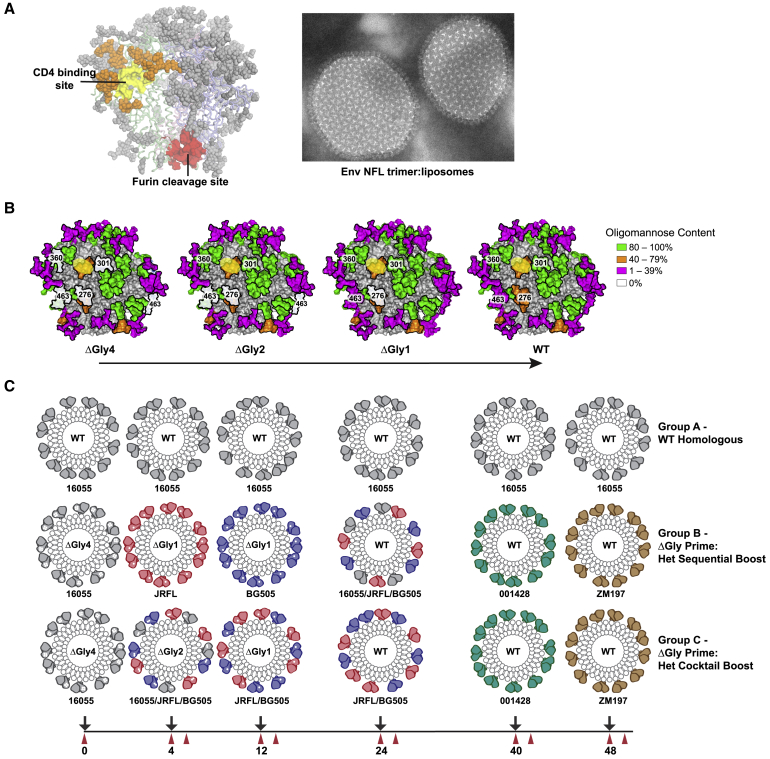
Immunogen Design to Facilitate ΔGlycan Prime:Heterologous Boost Strategies (A) Left: Env trimer model highlighting conserved sites of vulnerability, including the CD4bs (yellow) ringed with N-glycans (orange). Additional N-glycans masking the trimer are shown in dark gray. Right: NFL trimers coupled to liposomes imaged by nsEM. (B) Site-specific glycan analysis of the N-glycan-deleted immunogens compared to WT in the context of 16055 NFL TD CC^+^. Proximal N-glycans to the CD4bs (yellow) are indicated. Glycans are colored by oligomannose content with the deleted glycans in white. (C) Schematic overview of the immunogenicity regimen. 16055 (gray), JRFL (red), BG505 (blue), 001428 (teal), and ZM197M (tan) NFL trimer-liposomes formulated in adjuvant were administered subcutaneously in rabbits (6 per group) over a 48-week period, as noted by the black arrows. CD4bs-proximal N-glycan(s) were deleted (white circles) and used as priming immunogens in groups B and C. Bleeds were taken pre- and 2 weeks post-immunization as shown in red. See also Figure S1 and Table S1.

In selecting potential cross-conserved targets, we focused on the major protein surface required for viral entry and replication: the partially exposed CD4bs (Figure 1A, left). To encourage potential prime:boosting of both proximal and distal sites relative to the CD4bs, we merged multi-valent liposome array (Figure 1A, right) with (1) N-glycan deletion priming to better expose the underlying protein surface to B cells, followed by glycan restoration coupled with (2) heterologous Env trimer boosting to selectively drive B cells that could penetrate the intact N-glycan shield at the CD4bs or other conserved sites, while limiting boosting of autologous strain-restricted responses.

To generate the appropriate antigens, we utilized our “NFL TD CC^+^” design (Guenaga et al., 2015a, Guenaga et al., 2017) to produce trimers from diverse HIV Envs (i.e., 16055, BG505, JRFL, 001428, ZM197M). As previously shown, these cleavage-independent trimers are well-ordered, stable, homogeneous, and well-recognized by prototypic bNAbs (Guenaga et al., 2017), with cross-conserved sites at the V2 apex, the CD4bs, and the gp120:gp41 interface region as defined by sequence and bNAb recognition (Figure S2). A potential N-linked glycosylation site (PNGS) at position 332 was introduced in Envs naturally lacking N332 to maintain cross-conservation at the “N332 glycan supersite,” as needed (Dubrovskaya et al., 2017, Guenaga et al., 2017). To increase potential B cell responders to the CD4bs, both non-VRC01 and VRC01-class bNAbs directed to the CD4bs were considered in determining sites for genetic N-glycan deletion. As described previously, deletion of four PNGS sites (i.e., N276, N301, N360, N463) in the 16055 Env context increased recognition by CD4bs-directed bNAbs, while maintaining trimer stability and integrity, and resulted in greater neutralizing responses (Dubrovskaya et al., 2017). For this study, 16055 ΔGly4 was selected as the priming immunogen to initiate efficient B cell responses to the CD4bs. Based on this design, we also constructed ΔGly2 (ΔN276/463) and ΔGly1 (ΔN276) trimers in the 16055, JRFL, and BG505 Env contexts for the gradual restoration of the PNGS sites to be used in subsequent boosts to drive B cells that could penetrate the intact glycan shield (see Figures 1A and 1B). For the final boosts, we selected our most homogeneous and stable trimers that were well-recognized by bNAbs directed to the CD4bs and other conserved sites (Figures 1C and S1). NFL trimer 001428 was better recognized at the CD4bs and was used in the fifth inoculation, while ZM197M, being the most stringent “filter” at the CD4bs (but otherwise well-recognized at other conserved sites), was used in the last boost. All of the trimer immunogens contained C-terminal His tags for high-valency array on nickel-bearing liposomes (Figure 1A, right) as previously described (Dubrovskaya et al., 2017).

Analysis of the purified, fully glycosylated or glycan-modified trimers confirmed their structural integrity, antigenicity, and glycosylation profiles. No significant changes were detected in thermal melting temperatures (T_m_) between the wild-type (WT) and ΔGly trimers (Figure S1A). There was little difference in recognition by the conformationally sensitive trimer-specific bNAb PGT145 (V2 apex-directed) observed in the ΔGly trimers compared to WT (Figure S1B), confirming a native-like conformation, consistent with previous work (Dubrovskaya et al., 2017). We further assessed binding of the WT trimers against a panel of bNAbs directed to the CD4bs and other conserved neutralization sites to rank order those with the most favorable recognition profiles (Figure S1C). The PNGS genetic modifications were confirmed by mass spectrometry, and the remaining glycans were characterized by site-specific N-glycan analysis (Figures 1B and S1D; Table S1). Minimal local effects on glycan processing were detected at sites proximal to the genetic deletions when compared to WT equivalents (Figure S1D; Table S1). Removal of one or more glycan sites resulted in slightly increased processing at nearby sites, manifesting as a decrease in abundance of Man_9_GlcNAc_2_ and an increase in Man_8-5_GlcNAc_2_. The BG505 NFL CC^+^ trimer was the most sensitive to glycan deletion with more processing changes at other sites (Table S1), similar to what has been observed in glycan-depleted SOSIP trimers (Behrens et al., 2018). Following characterization, the NFL trimers were coupled to nickel-bearing liposomes (Bale et al., 2017, Ingale et al., 2016), and a high-density array was confirmed by negative stain EM (nsEM).

### N-Glycan Modification of NFL Trimers and Heterologous Prime:Boosting in Rabbits Elicit Cross-neutralization of Clinical HIV-1 Isolates

The overall immunization experiment is schematically shown in Figure 1C, performed over 48 weeks. Group A served as a “WT” control where animals were repeatedly inoculated with invariant, fully glycosylated 16055 NFL TD CC^+^ trimer-liposomes. To promote B cell responses toward the CD4bs, we used N-glycan deletion as well as WT trimers with more favorable binding profiles against CD4bs-targeted monoclonal antibodies (mAbs) earlier in the regimen (see Figure S1C). Both groups B and C assessed glycan deletion “priming” by 16055 ΔGly4 trimer-liposomes as the initial immunogen. To restore the glycans, we used two different approaches. Group B utilized sequential heterologous boosting, first with JRFL ΔGly1, then BG505 ΔGly1, followed by a boost with all three WT trimers arrayed as a cocktail on the same liposome for the fourth inoculation. Group C tested a more sequential glycan restoration approach using heterologous cocktails (arrayed on the same liposome) of first 16055/JRFL/BG505 ΔGly2 trimers, then JRFL/BG505 ΔGly1 trimers, followed by WT JRFL/BG505 trimers for the fourth immunization. After a long interval, the stabilized WT trimers, 001428 NFL TD CC^+^ and ZM197M NFL TD CC^+^, were used for the fifth and sixth inoculations, respectively (Figure S1C). All trimer-liposomes were formulated with ISCOMATRIX adjuvant prior to immunization.

**Figure 2:**
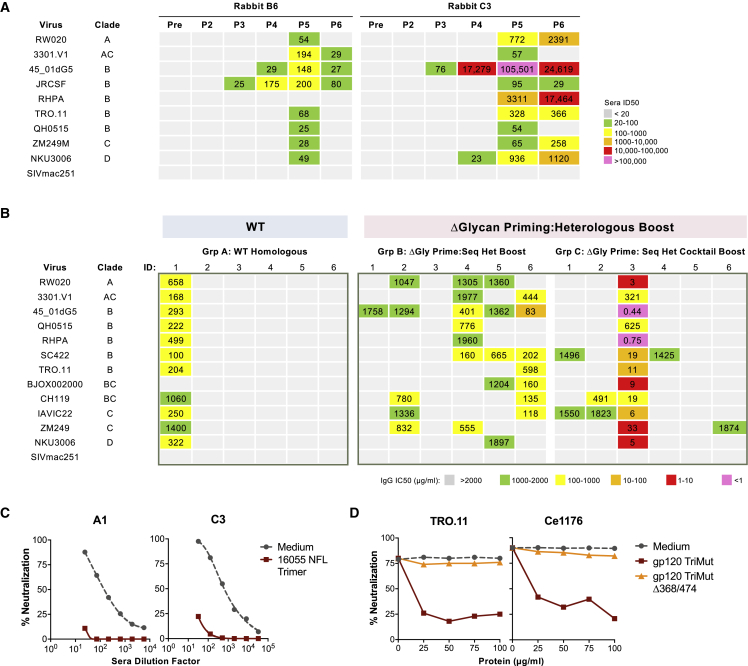
Serum and Purified IgG Cross-Neutralizes Clinical HIV-1 Isolates (A) Longitudinal cross-neutralization analysis, from pre-bleed (Pre) to post immunization six (P6), of serum from rabbits B4 and C3. ID_50_ (serum dilution at which 50% neutralization is achieved) values are colored as indicated. (B) Cross-neutralization of purified IgG (IC_50_, μg/mL, concentration at which 50% neutralization is achieved) from the post 6 time point against a panel of representative (tier 2) clinical HIV-1 isolates across different clades. (C) A1 and C3 post 6 sera were subjected to solid-phase adsorption using a 16055 NFL TD CC^+^ trimer affinity column to deplete Env binding Abs prior to neutralization assessment against virus TRO.11. A PBS (medium) lectin column was used as a negative control. (D) Differential adsorption of TRO.11 and Ce1176 virus entry. Purified IgG (post 5) from rabbit C3 were pre-incubated at a fixed concentration with culture medium (no inhibitor) or titrating amounts of the WT or CD4bs knockout (368/474) 16055 gp120 TriMut proteins prior to assessing neutralization against viruses TRO.11 and Ce1176. All neutralization assays were performed at least twice; representative data are shown. See also Figure S2.

As expected, we detected robust trimer-specific IgG binding responses from all animals following Env trimer-liposome inoculation (post 4–6) and weak anti-His responses (Figure S2A). Most animals from group A, which were immunized sequentially with 16055 NFL trimer-liposomes, developed autologous 16055 neutralizing responses (Figure S2B). We also detected weak neutralizing responses against viral strains used in the immunogen series from most animals in groups B and C, which received heterologous boosts, with rabbit C3 showing strong titers against four out of the five viruses (Figure S2B). Further analysis against panels of difficult-to-neutralize (“tier 2”) clinical isolates from different clades indicated serum cross-neutralizing responses in multiple animals following the fifth inoculation in groups B and C. Longitudinal analysis is shown for rabbits B6 and C3 (Figure 2A). We purified IgG from the serum of all rabbits and assessed cross-neutralization against a larger panel of viruses following the fifth and sixth inoculations (Figures 2B and S2C). The cross-neutralizing response was augmented post 6, where the number of responders increased, mostly in group B, with four out of six animals able to cross-neutralize at least five primary isolates. Within this group, rabbits B4 and B6 showed the highest neutralizing activity (Figure 2B, middle). Within group C, rabbit C3 displayed exceptional potency and breadth following both the fifth and sixth incolulations. One animal from group A (A1), which received only WT 16055 NFL TD CC^+^ trimer-liposomes, exhibited broad cross-neutralizing activity with less potency relative to C3 after the sixth immunization. Most of the cross-neutralizing activity was not apparent until post 6 for the group B rabbits as well as rabbit A1, whereas cross-neutralization was observed post 4 for rabbit C3. No neutralization was detected against the amphotropic viruses, SIVmac251 or SIVmac239, for any of the serum IgG samples, indicating HIV-1 Env specific neutralizing activity.

### Env-Specific Neutralization Maps Predominantly to the gp120 CD4bs from Antiserum-Derived IgG

To confirm HIV-1 Env specificity, we performed serum adsorptions using NFL trimers captured on *Galanthus nivalis* lectin-agarose beads as the “solid phase.” We used the V2-apex-directed bNAb, PGT145, as a positive neutralization control to confirm that such solid-phase trimers could deplete neutralization. As expected, PGT145 neutralizing activity of virus TRO.11 was substantially reduced by the trimer-lectin beads, but not by lectin beads alone (Figure S2D). Similarly, the neutralizing capacity of the A1 and C3 purified IgG from post 6 were substantially depleted by solid phase adsortion, confirming Env-specificity (Figure 2C).

We selected rabbit C3, which developed the most potent and broad neutralizing responses, for further epitope mapping. To determine whether any of the neutralizing activity was directed to the CD4bs, we used a differential adsorption assay comparing a CD4bs “knockout” mutant (D368R/M474A) to WT in the context of 16055 gp120 TriMut (altered to not bind CD4). As seen in Figure 2D, the IgG neutralizing activity from animal C3 against viruses TRO.11 and Ce1176 was greatly reduced after preincubation with the WT gp120 TriMut but not with the CD4bs knockout mutant, indicating CD4bs-directed activity. A marked reduction in neutralization activity was also observed in other viruses tested, including 16055 and X2278. Of note, not all activity was inhibited by the gp120 TriMut, as it did for the CD4bs-directed bNAb VRC13 positive control, indicating the possibility of other neutralizing activities that may not be gp120-directed (Figure S2E).

### Sorting of Hyperimmune Memory B Cells with Heterologous Env Probes Isolates HIV-1 Cross-Neutralizing mAbs

To identify and confirm the specificities mediating the observed HIV-1 cross-neutralization in rabbit C3, we utilized different sorting strategies to isolate single, live, Env-specific, IgG^+^ B cells from samples (i.e., lymph nodes, spleen, PBMCs) collected post 6 from rabbit C3 by fluorescence-activated flow cytometry (see Figure S3A; Tables S2–S4; STAR Methods). Heterologous Env probe pairs were used to enrich for cross-binding and potentially cross-neutralizing B cells. From matched heavy and light chains (HC and LC), we expressed the mAbs and screened for Env binding and neutralization against a small panel of viruses. While several only neutralized tier 1 (“lab-adapted”) strains, MN.3 and/or HXBc2, two mAbs, E70 and 1C2, exhibited cross-neutralizing activity against multiple tier 2 primary isolates and were selected for further analysis (Table S4). In terms of binding, 1C2 recognized all WT trimer immunogens with similar affinity, while notably E70 did not bind the JRFL NFL trimer immunogen (Figure S3B). Genetic analysis of the two Abs revealed their putative complementary determining regions (CDRs). However, because there is not a fully established database of expressed rabbit heavy and light chain repertoires, assignment of gene usage or somatic hypermutation (SHM) cannot be accurately determined for these mAbs. Nevertheless, based on the limited database in the International Immunogenetics Information System (IMGT) for rabbit Ig germline sequences, relevant features of these two mAbs are summarized in Figure S3C.

### mAb E70 Defines a Chimeric Glycan-Protein Cross-Neutralizing Determinant Proximal to the Conserved CD4bs

**Figure 3:**
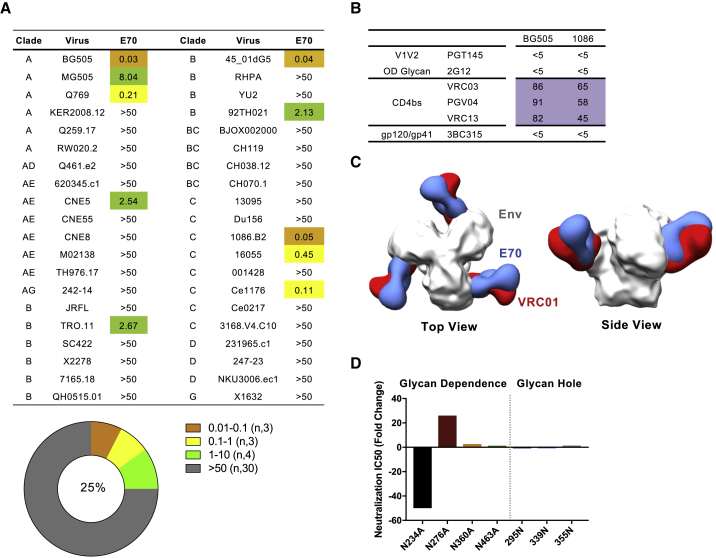
Vax**-**bNAb E70 Is Directed toward the CD4bs (A) Neutralization breadth and potency of E70 (IC_50_, μg/mL) against a 40-virus panel of (tier 2) HIV-1 clinical isolates. (B) Epitope mapping of E70 by cross-competition ELISA. Percent reduction of E70 binding to stabilized BG505 or 1086 NFL trimers in the presence of competing mAbs is indicated. (C) 3D reconstruction model of E70 Fab (blue) bound to BG505 NFL CC^+^ trimer (gray) by nsEM, shown with the CD4bs-directed VRC01 (red; EMD: 6252) docked in for reference. (D) Change in neutralization potency of E70 against a panel of glycan deleted or restored 16055 viruses. Neutralization and binding assays performed at least twice; representative data are shown. See also Figure S3 and Tables S2–S4.

To better determine E70 neutralization breadth, we screened a larger 40-virus panel encompassing multiple clades (Figure 3A). E70 neutralized 25% of the viruses with potencies ranging from 0.03 to 8.04 μg/mL. It neutralized all virus strains used for the Env trimer-liposome immunogens except for JRFL and 001428. To identify the binding specificity of E70, we performed a cross-competition ELISA with bNAbs to discrete Env sites and found that E70 cross-competed with the CD4bs-directed bNAbs (Figures 3B and S3D), suggesting that E70 was directed to this region. nsEM of E70 Fab in complex with the BG505 NFL CC^+^ trimer revealed binding toward the CD4bs but at an angle slightly different compared to the prototypic CD4bs-directed bNAb, VRC01 (Figures 3C and S3E). Accordingly, we analyzed E70 neutralization sensitivity relative to selected CD4bs-proximal N-glycans. As indicated in Figures 3D and S3F, deletion of the N-glycan at N234 renders the 16055 virus resistant to E70, which was corroborated with BG505 and 1086.B2 viruses possessing N234A or T236A mutations (Figure S3F). These results were consistent with JRFL being resistant to E70 as this Env naturally lacks the well-conserved glycan at residue N234. Restoration of this N-glycan in ZM249M (K236T) renders the virus sensitive to E70; however, N234 glycan restoration was not sufficient to confer JRFL sensitivity (Figure S3F). On the other hand, removal of glycan N276 increased neutralization potency and breadth against selected viruses (Figures 3D and S3F). These data indicate that the lack of glycan N234 alone is not the sole restriction to greater neutralization breadth and that the N276 glycan, either directly or indirectly, may impact on E70’s neutralizing capacity.

**Figure 4:**
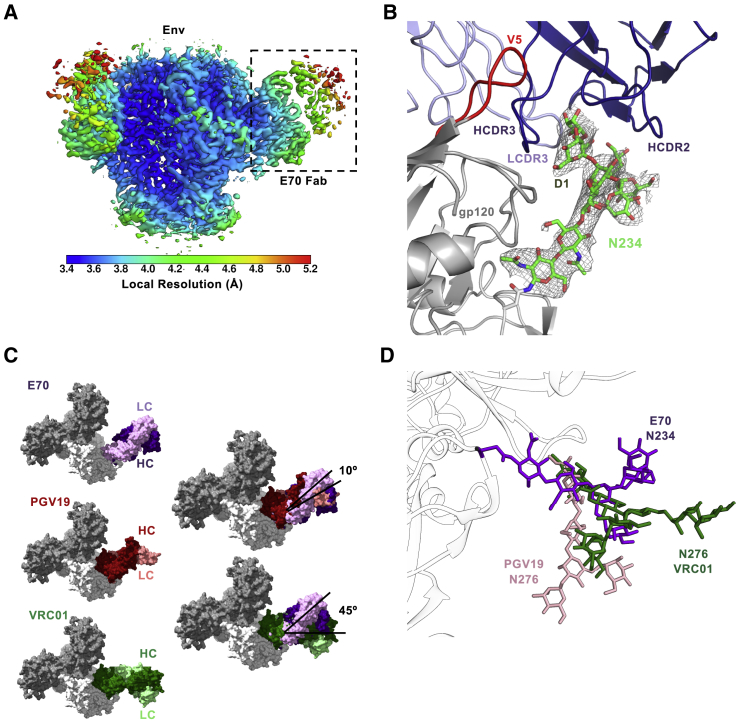
Cryo-EM Reconstruction of E70 with NFL Trimer Reveals a Glycan-Protein Cross-Neutralizing Epitope at the CD4bs (A) Cryo-EM reconstruction (∼3.7 Å resolution) of BG505 NFL CC^+^ trimer in complex with E70 Fab, colored by local resolution according to the key below. One Fab is boxed for reference. (B) E70 Fab interactions with N234 glycan, with glycan density displayed as a gray mesh (5σ contour). (C) Binding angle comparison of E70, VRC01 (PDB: 4LST), and PGV19 (PDB: 6B0N). gp120 subunits from the VRC01 and PGV19 structures were aligned to gp120 of the E70:BG505 complex model. (D) Relative position of N234 glycan (from E70:BG505 NFL CC^+^ trimer complex) compared to N276 from the VRC01 (PDB: 5FYK) and PGV19 (PDB: 6B0N)-bound Env complexes, respectively. See also Figure S4 and Table S5.

To better understand the intermolecular contacts between E70 and Env, we performed single-particle cryoelectron microscopy (cryo-EM) imaging of E70 Fab complexed with BG505 NFL CC^+^ trimer that resulted in an ∼3.7 Å resolution reconstruction (Figures 4A and S4A; Table S5). While the high-resolution structure further confirmed binding to the CD4bs, there was notably well-resolved density for glycan N234, showing primary interaction with the E70 HC and minor LC contacts (Figure 4B). The conserved N234 glycan (∼80%) is normally processed as an oligomannose with Man_9_ as the most common glycoform (Cao et al., 2018, Struwe et al., 2018). Although density was resolved only for a Man_5_ glycoform (including a complete D1 arm), a fully modeled Man_9_ can be accommodated by E70, as the D2 and D3 arms are distal to the HC (Figure S4B). The D1 arm of N234 is sequestered by a groove on E70 that is composed of all three heavy-chain complementarity-determining regions (HCDRs) and the light-chain complementarity-determining region 3 (LCDR3; Figures 4B and S4B), providing a major anchor point for the Ab, which helps explain the loss of neutralization potency against viruses lacking the N234 glycan. Another CD4bs glycan, N276, which is situated underneath glycan N234 parallel to the E70-gp120 interface, is not involved in binding in the BG505 context (Figure S4B). The epitope is completed with additional peptide contacts involving the HCDR3 of E70 and the C3 region of gp120 as well as interactions between LCDR1 and LCDR2 with the V5 loop of gp120 (Figures 4B, S4C, and S4D).

### Vaccine-Elicited E70 Approaches the Spike Similarly Compared to Human HIV Infection-Elicited CD4bs-Directed bNAbs

We compared E70 to different classes of patient-derived CD4bs-directed bNAbs, including PGV19 (VRC-PG19; (Zhou et al., 2013), VRC01 (Wu et al., 2010), CH103 (Liao et al., 2013), and CH235 (Bonsignori et al., 2016). Although compared to the other bNAbs, E70 is rotated ∼180° with the LC on top when viewed from the trimer apex and the HC below, E70 approaches the CD4bs at similar angles as PGV19 and CH235 (Figures 4C and S4E). Meanwhile, CH103 and VRC01 approach at a more outward angle from the interprotomer interface. While further comparison between E70, PGV19, and VRC01 shows some direct epitope overlap between all three Abs (∼20% of specific contact residues) (Figure S4F), the Abs also differ in their interactions with specific, but proximal, N-glycans. Both VRC01 and PGV19 interact with (but are not dependent on) glycan N276, whereas E70 engulfs the D1 arm of the N234 glycan, required for binding and neutralization. In contrast, PGV19 and VRC01 contact the glycan base through core GlcNac and β-Man residues, displaying less dependence on the branching D1 and D2 arms (Figure S4G). An overlay of the primary glycan contact from all three Ab complexes reveals substantial structural overlap in three-dimensional space, suggesting that the N234 and N276 glycans may, in some cases, be interchangeable for eliciting an Ab response against the CD4bs (Figure 4D). Both glycans, however, cannot be engaged by a single Ab, at least by those described here.

### The 1C2 bNAb Binds to the gp120:gp41 Interface and Displays Env-Destabilizing Properties

**Figure 5:**
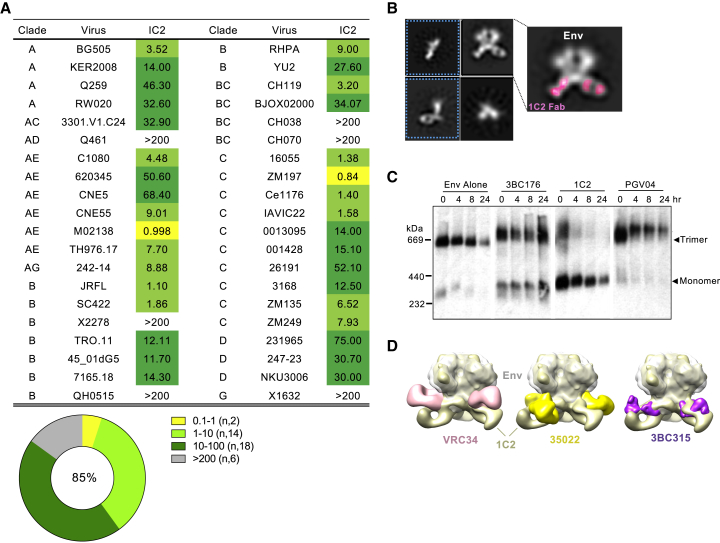
Broadly Neutralizing mAb 1C2 Binds the gp120:gp41 Interface and Destabilizes the Env Trimer (A) Neutralization profile of 1C2 against a panel of (tier 2) HIV-1 clinical isolates with IC_50_ (μg/mL) values as indicated. (B) nsEM analysis of 1C2 bound to 16055 NFL TD CC^+^ trimer. Representative 2D class averages are shown with 1C2 highlighted in pink and dissociated trimers boxed in blue. (C) BG505 virions were pre-incubated at 37°C alone or with 20 μg/mL 3BC176, 1C2, or PGV04 for various time periods, after which Env was solubilized, separated using BN-PAGE, and visualized by western blot using gp120 and gp41-specific Abs. Trimer and gp120:gp41 monomer bands are indicated. (D) 3D reconstruction models of 1C2-bound 16055 NFL TD 2CC^+^ trimer, which includes an additional disulfide bond that prevents trimer dissociation, from nsEM analysis. Structures of human Fabs directed toward the gp120:gp41 interface were aligned to the C3 refined map and docked in for comparison against 1C2: VRC34 (pink, FP targeting; EMD: 8125), 35O22 (yellow; EMD: 2672), and 3BC315 (purple; EMD: 3067). See also Figures S3 and S5 and Table S4.

We next focused on the other isolated cross-neutralizing Ab, 1C2. Analysis against a 40-virus panel revealed moderate potency but greater breadth than E70, with 85% of the viruses neutralized (Figure 5A). To determine binding specificity, we performed cross-competition ELISAs, but no cross-competition was detected against bNAbs to the trimer apex V1V2, the CD4bs, or the N332 glycan supersite (Figure S5A). Further analysis revealed that 1C2 bound to gp140 but not gp120 (Figure S5B). To pinpoint 1C2 binding specificity, we performed nsEM using 1C2 Fab in complex with the 16055 NFL TD CC^+^ trimer, which indicated the gp120:gp41 interface as the target. Further analysis was complicated due to apparent dissociation of the Env trimer, as a large population of 1C2-bound gp140 monomers was observed (Figure 5B). Dissociation was also seen using other Env strains, whereas no effect was observed with other Fabs (e.g., VRC01). This putative ability of 1C2 to destabilize the trimer is reminiscent of the human bNAbs 3BC315 and 3BC176, both of which target the gp120:gp41 interface (Lee et al., 2015).

To test whether 1C2-mediated dissociation of the trimer might be relevant to the mechanism of neutralization, we tested its effect on Env stability on the viral surface. 1C2 Fab was incubated with virus at 37°C. The 1C2-Env complexes were solubilized from the membrane and resolved by native gels. 3BC176 and PGV04 were used as positive and negative controls. While the trimer remained intact with PGV04, it dissociated into gp120:gp41 monomers in both the 3BC176 and 1C2 preparations (Figure 5C). We also tested 1C2′s ability to destabilize functional Env using a pre-incubation neutralization assay (Lee et al., 2015). While the standard assay measures an antibody’s neutralization activity after a 1-h preincubation with pseudovirus, this assay determines the activity over a 24-h time period to quantitate irreversible destabilization. As observed for both JRFL and BG505 viruses, greater apparent neutralization activity was detected at 24 h compared to 1 or 6 h for 1C2, 3BC176, and 3BC315, whereas the activity remained relatively unchanged for PGV04 and T20 (fusion inhibitor) (Figure S5C). Together, these data implicate 1C2 as having a similar Env destabilizing activity as 3BC315 and 3BC176.

### Vaccine-Elicited 1C2 Is Similar to the Human bNAb 3BC315 but with Greater Neutralizing Breadth

To facilitate structural studies in delineating 1C2′s epitope, we screened additional stabilized Env trimers. An inter-protomer cysteine disulfide linkage (501C-663C; Yang et al., 2018) prevented 1C2-mediated dissociation by native gel analysis (Figure S5D). The enhanced stability permitted 3D reconstructions of the 1C2:16055 NFL TD 2CC^+^ complex by nsEM (Figure S5E). 1C2 again has the closest resemblance to 3BC315 compared to other interface bNAbs but with the Fabs rotated 90° relative to each other (Figures 5D and S5E). By cross-competition, 1C2 competed with 3BC315 but not VRC34 (Figure S5F).

**Figure 6:**
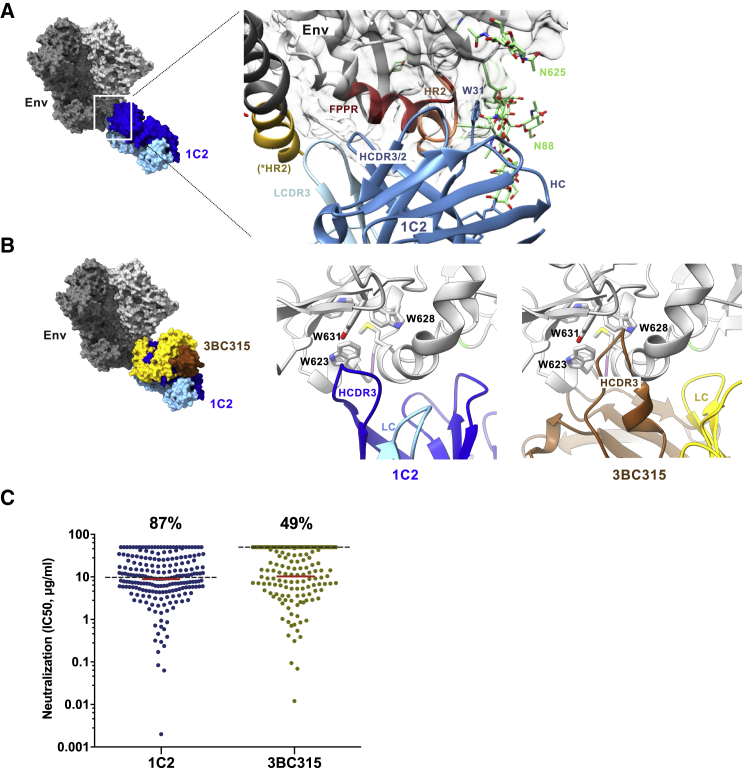
Vaccine-Elicited bNAb 1C2 Is Similar to the Human bNAb 3BC315 (A) Reconstruction of 1C2 (blue) in complex with 16055 NFL TD 2CC^+^ Env trimer from the ∼3.9 Å resolution cryo-EM structure. Magnified view of the epitope is shown to the right. The 1C2 HC framework regions interact with glycan N88 and stabilize in a position close to glycan N625. The HCDRs and LCDR3 are involved in peptide contacts with gp41, primarily around the fusion peptide proximal region (FPPR) and HR2 helix. HCDR3 of 1C2 is near the tryptophan clasp of gp41, marked by residue W31. ^∗^HR2, from adjacent protomer. (B) The medium resolution cryo-EM reconstruction of 3BC315 Fab in complex with BG505 SOSIP trimer (EMD: 3067) was aligned to the map of the 1C2-bound trimer complex. The docked crystal structure of 3BC315 Fab (PDB: 5CCK) is shown relative to 1C2. Magnified views of 1C2 interaction with the tryptophan clasp of gp41 are shown to the right compared to 3BC315. (C) Neutralization potency (IC_50_, μg/mL) of 1C2 compared to 3BC315 in a 208-virus panel. Percentage of neutralized viruses are indicated at the top. Median IC_50_ of all viruses (gray dashed line) compared to sensitive viruses only (red line; non-neutralized viruses excluded) are shown. See also Figures S6 and S7 and Tables S5 and S6.

Next, we obtained a high-resolution cryo-EM structure of the 1C2:16055 NFL TD 2CC^+^ complex at ∼3.9 Å resolution (Figure S6A; Table S5). The high-resolution crystal structure of 1C2 Fab (Figure S6B; Table S6) was used as a starting model. The Ab primarily contacts gp41 and the N88 glycan of gp120 (Figure 6A). Compared to other high-resolution Env trimer models, glycan N88 in the 16055 NFL TD 2CC^+^ trimer is repositioned by the interaction with 1C2, which in turn, repositions glycan N625 (Figure S6C). By independent analyses, N88 is not required for 1C2 recognition as the neutralization potency of 1C2 increases with removal of the glycan in N88A viral mutants (Figure S6D). The cryo-EM-derived model reveals that 1C2 can accommodate the N88 glycan with primary contacts between W31 of HCDR1 and the glycan base as well as polar interactions between the HC framework 3 residue R94 and a branching arm sugar (Figure 6A). Primary peptide contacts involve both HCDR2 and HCDR3 with parts of the fusion peptide proximal region (FPPR) and HR2 of gp41, while secondary contacts involve LCDR3 of 1C2 (Figure 6A).

Given the similarities between 1C2 and 3BC315, we compared their angles of approach and epitopes more closely. While a high-resolution model of 3BC315 in complex with trimer is unavailable, we fitted the 1C2:16055 NFL TD 2CC^+^ reconstruction into the published ∼9 Å resolution reconstruction of 3BC315:BG505 SOSIP (EMDB: 3067) and then docked the 3BC315 Fab crystal structure (PDB: 5CCK). From this model, 3BC315 approaches the spike at an angle more parallel to the viral membrane, whereas 1C2 is closer to the membrane (Figure 6B). Nevertheless, 1C2 can effectively neutralize native viruses and bind to Env on the cell surface (Figure S6E). Removal of N-glycans N88 and N625 improved 1C2 cell-surface binding, consistent with glycan impediment (Figure S6E).

Similar to 1C2, 3BC315 has increased neutralization potency against N88A viral mutants (Figure S6D; Lee et al., 2015). When comparing the positions of the N88 and N625 glycans in the 1C2-bound trimer, the HC of 3BC315 would clash with the N88 glycan, suggesting a different mode of interaction compared to 1C2. One proposed neutralization mechanism by 3BC315 is via disruption of the tryptophan clasp domain of gp41, a conserved region of mainly aromatic residues that confers stability in both the unliganded prefusion and receptor-bound prefusion intermediate states of Env (Ozorowski et al., 2017, Pancera et al., 2014). The cryo-EM reconstruction of 1C2-bound Env reveals that the Ab surrounds the FPPR of gp41 and the HCDR3 wraps underneath toward the tryptophan clasp residues (Figure 6B, middle). Fitting of the 3BC315-bound and 1C2-bound maps suggests that 3BC315 also interacts in a similar manner with the HCDR3 of 3BC315 penetrating even deeper into the tryptophan clasp (Figure 6B, right). Of note, the extra stability conferred by the additional disulfide bond introduced in the 16055 NFL TD 2CC^+^ trimer used for the 1C2 cryo-EM complex may have prevented full disruption of the tryptophan clasp and thereby the trimer.

We also compared the neutralization capacity of 1C2 to 3BC315 in a 208-virus panel (Figures 6C and S7), revealing greater breadth of 1C2 at 87% compared to 49% for 3BC315 (IC_50_ at <50 μg/mL). Accounting for viruses neutralized at an IC_50_ <200 μg/mL, breadth increased to 92% for 1C2 and 61% for 3BC315. Both have similar median IC_50_ values for viruses neutralized at <50 μg/mL (1C2, 8.9 μg/mL; 3BC315, 10 μg/mL). In sum, the vaccine-elicited rabbit bNAb 1C2 shares many similarities with the infection-elicited human bNAbs 3BC315/3BC176 but achieves greater neutralization breadth. 1C2 is also broader compared to the recently described vaccine-elicited class of mAbs directed to the FP, including DFPH-a.01, which neutralizes 59% of the 208-virus panel (Kong et al., 2019). Further comparison between 1C2 and DFPH-a.15 reveals both mAbs binding to a similar region but with 1C2 away from the FP (Figure S6F).

### mAbs Derived from Rabbit C3 Reconstitute Most of the Serum IgG-Mediated Neutralization

**Figure 7:**
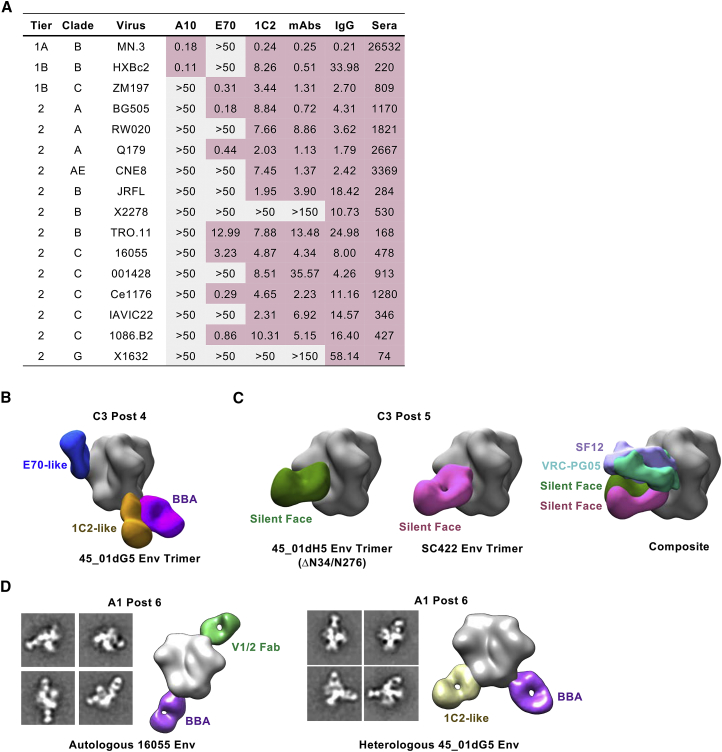
Epitope Mapping of Cross-Neutralizing Responses from Multiple Animals Reveals Similar Recognition of Cross-Neutralizing Determinants (A) Neutralization profile of select mAbs isolated from animal C3 (A10, E70, 1C2), assessed individually or together (“mAbs”), compared to total purified IgG and serum (post 6) against a 16-virus panel. IC_50_ (μg/mL) values are indicated for the mAbs and purified IgG; ID_50_ values shown for the sera. Red, neutralized; gray, not neutralized. (B) 3D reconstruction model of purified IgG Fabs from animal C3 at the post 4 time point complexed with heterologous trimer 45_01dG5 NFL TD 2CC^+^. Predominant Fab populations are shown for the E70-like (blue), 1C2-like (brown), and base-binding antibody (BBA, purple). (C) Reconstruction models of purified IgG Fabs from rabbit C3 at post 5 complexed with heterologous trimers 45_01dH5 NFL TD 2CC^+^ (left), which naturally lacks glycans N234 and N276, or SC422 NFL TD CC^+^ (middle). The predominant Fab populations directed to the silent face are shown (green and pink). Right, patient-derived bNAbs, SF12 (lavender), and VRC-PG05 (teal) were docked in for comparison. (D) EM analysis of sera-derived IgG Fabs from rabbit A1 post 6 complexed with autologous 16055 NFL TD CC^+^ trimer (left) or heterologous 45_01dG5 NFL TD 2CC^+^ trimer (right). Representative 2D class averages are shown with the 3D reconstruction model. Fabs directed to V1V2 (light green) and the trimer base (BBA, purple) were predominant against 16055, while only 1C2-like (tan) and base-binding (BBA, purple) were detected with the 45_01dG5 trimer.

To confirm whether the isolated mAbs account for the observed breadth of animal C3, we compared the neutralization profile of mAbs A10, E70, 1C2 (alone and together) with the purified IgG and sera (Figure 7A). The purified IgG and sera neutralized all viruses tested in the panel. The selected mAbs alone (or mixed together as a cocktail) accounted for most but not all of the neutralizing activity detected with the purified IgG or sera, with most of the observed breadth attributable to 1C2 and/or E70.

### nsEM of the Serum-Derived Fabs from Rabbits C3 and A1 Reveals Targeting of Multiple Cross-Recognized Epitopes

Employing an alternative yet complimentary approach, we utilized EM polyclonal epitope mapping of serum Abs developed in the Ward and Hangartner groups (Bianchi et al., 2018) to identify the major sites targeted by the serum Abs. Our main objective was to define cross-neutralizing serum IgG specificity using heterologous Envs (not in the immunogen series) and track their development in a longitudinal manner. Because neutralization of virus 45_01dG5 at the post 3 to post 4 time points was one of the early indicators of developing cross-neutralizing activity in animal C3, we used 45_01dG5 NFL TD 2CC^+^ trimers to map this activity. We generated Fabs from purified C3 IgG, incubated with the trimer, and imaged the immunocomplexes by nsEM. From the post 4 sample, we were able to detect Fab densities targeting both the CD4bs and the gp120:gp41 interface, likely representing E70-like and 1C2-like Fabs, respectively (Figure 7B). We also detected other base-binding Abs (BBAs) directed to the tip of HR2 and the FPPR, approaching from different angles (Figure 7B). To determine whether there are other cross-neutralizing CD4bs-directed Abs besides the N234 glycan-dependent E70, we used the related 45_01dH5 NFL TD 2CC^+^ trimer that naturally lacks the N234 glycan to assess post 5 samples when more cross-neutralizing activity was apparent. We did not detect any Fabs directed toward the CD4bs with the N234-glycan deficient trimer, indicating that E70 likely represents the major class of cross-recognizing Ab to this site. In the absence of E70 interaction, we now detected a new density to the so-called trimer “silent face” (i.e., backside of the CD4bs) (Figure 7C), another human bNAb target as exemplified by VRC-PG05 and SF12 (Schoofs et al., 2019, Zhou et al., 2018). Similar silent face Fabs were also identified using another Env strain (SC422) that can be neutralized by C3 sera/IgG, suggesting a possible third cross-neutralizing specificity elicited in this rabbit. 1C2-like Fab densities were not readily identified in this post 5 sample, which may have been outcompeted by the increased population of BBAs at this time point.

We also utilized this technique to map the potential neutralizing specificities present in the serum IgG of rabbit A1, which displayed relatively broad but not as potent neutralization following six inoculations with the 16055 NFL TD CC^+^ trimer-liposomes. We first performed the polyclonal Fab:nsEM analysis with the 16055 NFL TD CC^+^ “autologous” trimer used in the immunogen and detected densities targeting the V1/V2 apex and the base of the trimer (Figure 7D). The targeting of the V1/V2 region of 16055 is consistent with our previous isolation of autologous NAbs directed to this region (Martinez-Murillo et al., 2017). To map cross-neutraliazation, we again used the 45_01dG5 trimer and detected only a 1C2-like density against the interface, suggesting that the serum cross-neutralization from rabbit A1 also maps to this functionally conserved region, similar to the 1C2 bNAb from rabbit C3.

## Discussion

In this study, we analyzed multiple means of presenting the well-ordered NFL trimers arrayed at high density on synthetic liposomal particles to assess their ability to induce cross-neutralizing Abs. We focused on boosting conserved protein determinants to promote cross-neutralizing responses by (1) utilizing Δglycan priming to increase B cell accessibility, (2) glycan restoration to selectively boost B cells that can access the CD4bs, (3) heterologous boosting to drive cross-conserved epitopes, and (4) liposomal multi-valent particulate array to increase B cell activation. Together these strategies successfully elicited cross-neutralizing responses in multiple animals, primarily in the Δglycan-primed subjects. From the Δglycan-primed rabbit displaying the broadest and most potent neutralizing responses, we isolated two vaccine-elicited bNAbs (vax-bNabs), E70 and 1C2, directed to two distinct sites of Env vulnerability. While polyclonal mapping of the sera-derived Fabs by nsEM confirmed these two targeted sites in rabbit C3, a potential third site to the Env silent face was also detected. To have two, possibly three, sites targeted is very encouraging for this approach, increasing the possibility to elicit cross-neutralizing Abs by vaccination.

The vax-bNAb, E70, is directed to the CD4bs and displays 25% breadth against a panel of clinical isolates. As infection-elicited, CD4bs-directed mAbs can range in breadth from 3% (VRC40) to 36% (HJ16) and up to 98% (N6), the elicitation of E70 is an encouraging initial result. The high-resolution structure shows that the epitope consists of roughly 50% “self” N-glycan and conserved polypeptide surface to present a chimeric protein:glycan epitope that is seen as foreign by mammalian B cells. Our study provides proof of principle that targeting of the N-glycan shield is possible following trimer vaccination and might be more efficient using targeted glycan-deleted Env immunogens, as recently shown for priming at the N332 supersite (Escolano et al., 2019). Based on our studies and others who have shown improved neutralizing responses using targeted glycan deletion (Crooks et al., 2017, Dubrovskaya et al., 2017, Ringe et al., 2019, Zhou et al., 2017), presenting Env in ways not seen by HIV during natural infection, such as site-specific deglycosylation to “de-evolve” the glycan shield and thereby altering the elicited responses evolved against it, represents a viable strategy going forward. E70 is also distinct from other CD4bs-directed bNAbs like PGV19 and VRC01 because it engages an entire branching arm of the 234 glycan, which may play a role in limiting potential breadth. For PGV19 and VRC01, primary contacts are at the base of the glycan, typically involving core GlcNac and β-Man sugars with less dependence on the branching arms, which might be required due to heterogeneity in N276 glycan processing. Recent mass spectrometry analysis suggests genotype-dependent variability in the mixture of complex and oligomannose glycoforms at position N276, as well as heterogeneity in the types of oligomannose forms (Cao et al., 2018, Struwe et al., 2018). Abs that evolve to the glycan base can therefore better accommodate glycan heterogeneity without impacting potential breadth.

The other isolated vax-bNAb, 1C2, is directed to the gp120:gp41 interface and is a very broad mAb elicited by repeated vaccination with the liposome-presented cleavage-independent trimers. The elicitation of 1C2, together with the recent success in eliciting bNAbs against the FP (Xu et al., 2018), illustrates that the general region proximal to the gp120:gp41 interface is a viable target and that both NFL and SOSIP trimer designs may have utility in elicitating Abs to this epitope area. Identification of multiple bNAbs to this region, typified by 3BC315 (interface) and VRC34 (FP), may in part be facilitated by the less dense N-glycan coverage in this area to allow access by cellular furins as well as functional gp41 conservation. The FP is less exposed in the NFL trimers since this motif is covalently linked to gp120 in the NFL design, as indicated in the crystal structure (Sarkar et al., 2018), perhaps rendering the FPPR, HR1, and HR2 more immune attractive to B cells than the FP. The lack of direct association with the FP may also contribute to the increased breadth of 1C2, rendering it less sensitive to FP sequence variation. The trimers used here are well recognized by the interface-directed bNAbs, 3BC315 and 35O22, a consideration for re-elicitation of 1C2-like Abs. Since the CC2 inter-protomer cysteine disulfide linkage (501C-663C) helps to stabilize this region in the NFL context, it can be considered for immunogen redesign. The stabilizing CC2 disulfide, coupled with particulate display of alternative trimer orientations and prime:boosting using different trimer types, may better target this naturally glycan-sparse region.

Besides E70- and 1C2-like, we also detected other cross-binding Fab specificities in rabbit C3. When we used an Env that naturally lacks the N234 glycan, abrogating E70 recognition, Fabs directed to the gp120 silent face were revealed by nsEM. This Fab density was more readily detectable in the absence of E70-like (or CD4bs-directed) activity, presumably due to removal of steric hindrance to this epitope on the adjacent protomer. Whether silent face-directed Abs contribute to cross-neutralization remains to be determined.

Broad neutralization also developed in rabbit A1. Epitope mapping indicated that the cross-neutralizing activity is likely directed to the Env interface, similar to 1C2. It will be interesting to determine whether the cross-neutralizing responses seen in the other animals (e.g., B4 and B6) also target the interface or other conserved elements. That rabbit A1, which only received 16055 NFL trimer-liposome inoculations, developed cross-neutralizing activity at the interface over time is encouraging, as simplified regimens using fewer different Env trimers may be possible.

Both rabbits C3 and A1 also generated BBAs, as indicated by nsEM. While the neutralizing activity of the BBAs remains to be determined, coupling the trimer immunogens at the base to liposomes, as utilized in this study, may help reduce non-neutralizing, base-directed responses.

The trimeric immunogens used in this study all contain C-terminal His tags for liposomal coupling. We have evaluated the NFL trimers both with and without the His tag in previous studies (Dubrovskaya et al., 2017, Guenaga et al., 2015a, Sarkar et al., 2018), and both formats demonstrated very similar biophysical properties (i.e., thermal stability or trimeric homogeneity as determined by EM) and antigenic profiles. The high-resolution EM structures presented here, which utilized 16055 and BG505 NFL trimers with His tags, are comparable to the crystal structures that used 16055 and BG505 NFL trimers lacking His tags (Guenaga et al., 2015b, Sarkar et al., 2018) to favor crystal packing. We have shown previously that the His-to-nickel linkage can dissociate under *in vivo* conditions, eliciting responses to the His tag following multiple trimer-liposome immunization (Bale et al., 2017), which is consistent with the weak anti-His responses detected here. Decreasing anti-His responses alone, by utilizing covalently coupled trimer-liposomes, did not favor the elicitation of NAbs in mice. Removal of the His tag would be considered for clinical development, although His-tagged proteins are used clinically (e.g., Blinatumomab).

Despite the presence of potentially non-neutralizing BBAs and anti-His Abs, multiple rabbits in this study were able to develop cross-neutralizing responses, including those directed to the gp120:gp41 interface. Whether elimination of anti-His and/or non-neutralizing base responses in general would favor the elicitation of cross-neutralizing activity remains to be determined.

In the prime:boost regimens explored here, there are multiple means to improve upon the affinity, neutralizing potency, and frequency of the responses. Although mapping by both EM and virus neutralization provide insight as to when a given neutralizing specificity becomes detectable in the serum, it is not yet clear exactly when each specificity primes or how critical each Env sequence is for each prime or boost. Unlike other approaches that are more prime dependent (e.g., germline-reverted bNAb targeting), *de novo* responses, in principle, can be primed throughout the regimen and are not necessarily dependent on previous immunizations to activate a given B cell lineage. This may be why cross-neutralization is not readily detected until the fifth or sixth immunization in this experiment or that it simply takes several months for affinity maturation of cross-reactive B cell responses. The cross-neutralization developed later in A1, suggesting that given enough time and/or repeated exposure to Env, the number of different strains may be reducible. However, to increase frequency, such simple regimens will need to be further augmented, perhaps by incorporating elements from the Δglycan prime:boost strategy utilized in group B and applied to other conserved sites (e.g., trimer interface, N332 glycan super site) or by other approaches. Conversely, whether the group B/C regimens can be simplified, while improving response quality, robustness, frequency, and translatability, merits further exploration. Ultimately, both breadth and potency are likely necessary for an effective vaccine, and optimization of the responses obtained here will be needed for robust protection. Longer regimens may help SHM and increase potency by increasing affinity for the native spike. A vaccine that elicits responses against multiple epitopes could also help increase breadth, potency, and effectiveness overall, akin to the development of bi- and tri-specific Abs.

Here, we selected rabbits because this animal model is well proven for vaccine assessment; however, as with any animal model, there are caveats to be considered. Rabbits differ from humans in their gene segment sequences, express limited VH alleles, and use gene conversion to generate diversity in roughly 23% of their Ig repertoire (Lavinder et al., 2014). However, as outbred animals they can better model the difficulty of generating Abs against HIV following Env vaccination in a diverse population such as humans. We show here that rabbits can generate bNAbs targeting epitopes by similar means of recognition compared to those generated by the human immune system, indicating that they are a relevant animal model for proof-of-principle testing.

In summary, one vax-bNAb is directed to the CD4bs (E70) and the other to the gp120:gp41 interface (1C2). At the core of these respective epitopes are conserved protein determinants ringed by glycans that are required to be exposed for Env function and virus survival. The functionally conserved CD4bs is necessary for receptor interaction, and the interface for furin accessibility, cleavage, and the functional capacity to mediate fusion and viral entry. Both Abs imitate human HIV-infection-induced bNAbs in their general means of accessing their glycan-shrouded epitopes on the trimer. These results validate epitope targeting and the “surface-presentation” approach. The cross-neutralizing responses detected in multiple animals to different epitopes is an encouraging first step to more efficiently elicit cross-neutralization of clinical isolates at different sites of vulnerability following Env vaccination.

## STAR★Methods

### Key Resources Table

**Table:** 

REAGENT or RESOURCE	SOURCE	IDENTIFIER
**Antibodies**		

Donkey anti-rabbit IgG (H+L)-FITC	Jackson ImmunoResearch	Cat#711-095-152; RRID: AB_2315776
Mouse anti-His tag	R&D Systems	Cat#MAB050; RRID: AB_357353

**Chemicals, Peptides, and Recombinant Proteins**		

16055 NFL TD CC+ trimer	Guenaga et al., 2017	N/A
16055 ΔGly4 NFL TD CC+ trimer	Dubrovskaya et al., 2017	N/A
16055 ΔGly2 NFL TD CC+ trimer	This manuscript	N/A
16055 ΔGly1 NFL TD CC+ trimer	This manuscript	N/A
JRFL NFL TD CC+ trimer	Guenaga et al., 2017	N/A
JRFL ΔGly2 NFL TD CC+ trimer	This manuscript	N/A
JRFL ΔGly1 NFL TD CC+ trimer	This manuscript	N/A
BG505 NFL CC+ trimer	Guenaga et al., 2017	N/A
BG505 ΔGly2 NFL CC+ trimer	This manuscript	N/A
BG505 ΔGly1 NFL CC+ trimer	This manuscript	N/A
001428 NFL TD CC+ trimer	Guenaga et al., 2017	N/A
ZM197M NFL TD CC+ trimer	Guenaga et al., 2017	N/A
16055 NFL TD 2CC+ trimer	Yang et al., 2018	N/A
SC422 NFL TD CC+ trimer	Guenaga et al., 2017	N/A
45_01dG5 NFL TD 2CC+ trimer	This manuscript	N/A
45_01 dH5 NFL TD 2CC+ trimer	This manuscript	N/A
Galanthus nivalis lectin-agarose	Vector Laboratories	Cat#AL-1243
DGPC	Avanti Polar Lipids	Cat#850365
DGS-NTA(Ni)	Avanti Polar Lipids	Cat#790404
Cholesterol	Sigma	Cat#C3045
Ficoll-Paque PLUS	GE Healthcare	Cat#17-440-03
293fectin	Life Technologies	Cat#12347500
Biotinylated wheat germ agglutinin	Vector Laboratories	Cat#B-1025
3,3′5,5;-tetramethylbenzidine (TMB) substrate	Life Technologies	Cat#002023
Random hexamers	Gene Link	Cat#26-4000-03
RNaseOut	Life Technologies	Cat#10777019
FreeStyle 293 expression medium	Life Technologies	Cat#12338026
rProtein A Fast Flow	GE Healthcare	Cat#17127903
Superscript III Reverse Transcriptase	Life Technologies	Cat#56575
Superscript IV Reverse Transcriptase	Life Technologies	Cat#18090010
Phusion Hot Start II High Fidelity DNA Polymerase	Life Technologies	Cat#F549L
HotStar Taq Plus DNA Polymerase	QIAGEN	Cat#203603
NEBuilder High Fidelity DNA Assembly Master Mix	New England Biolabs	Cat#E2621L
HRP-conjugated streptavidin	Sigma	Cat#S2438
n-Dodecyl-β-D-Maltopyranoside (DDM)	Anatrace	Cat#D310
Uranyl Formate	Electron Microscopy Sciences	Cat#22451

**Critical Commercial Assays**		

Advanced Protein Assay Reagent	Cytoskeleton	Cat#ADV01-A
QuikChange Multi-site Lightning Kit	Agilent	Cat#210513
BirA biotin-protein ligase standard reaction kit	Avidity	Cat#BirA500
Negative stain EM Grids	Electron Microscopy Sciences	Cat#EMS400-CU
CryoEM grids	Electron Microscopy Sciences	Cat#Q26194

**Deposited Data**		

CryoEM map of E70 Fab in complex with BG505 NFL CC+	EMDataBank	EMD: 20259
	RCSB Protein Data Bank	PDB: 6P62
CryoEM map of 1C2 Fab in complex with 16055 NFL TD 2CC+	EMDataBank	EMD: 20260
	RCSB Protein Data Bank	PDB: 6P65
Negative stain EM reconstruction of E70 Fab in complex with BG505 NFL CC+	EMDataBank	EMD: 20273
Negative stain EM reconstruction of 1C2 Fab in complex with 16055 NFL TD 2CC+	EMDataBank	EMD: 20274
Negative stain EM reconstruction of polyclonal serum from rabbit C3 at Post 4 in complex with 45_01dG5 NFL TD 2CC+	EMDataBank	EMD: 20343
Negative stain EM reconstruction of polyclonal serum from rabbit C3 at Post 5 in complex with 45_01dH5 NFL TD 2CC+	EMDataBank	EMD: 20279
Negative stain EM reconstruction of polyclonal serum from rabbit C3 at Post 5 in complex with 45_01dH5 NFL TD 2CC+ (silent face Fabs only)	EMDataBank	EMD: 20280
Negative stain EM reconstruction of polyclonal serum from rabbit C3 at Post 5 in complex with SC422 NFL TD CC+	EMDataBank	EMD: 20342
Negative stain EM reconstruction of polyclonal serum from rabbit A1 at Post 6 in complex with 45_01dG5 NFL TD 2CC+, class 1	EMDataBank	EMD: 20347
Negative stain EM reconstruction of polyclonal serum from rabbit A1 at Post 6 in complex with 45_01dG5 NFL TD 2CC+, class 2	EMDataBank	EMD: 20346
Negative stain EM reconstruction of polyclonal serum from rabbit A1 at Post 6 in complex with 16055 NFL TD CC+	EMDataBank	EMD: 20345
X-ray crystal structure of 1C2 Fab	RCSB Protein Data Bank	PDB: 6PEH

**Experimental Models: Cell Lines**		

Human: FreeStyle 293F	Invitrogen	Cat#R79007
Human: TZM-bl	NIH AIDS Reagent Program	Cat#8129
Human: 293T	ATCC	Cat#CRL-3216

**Experimental Models: Organisms/Strains**		

Rabbit: New Zealand White	Covance Research Products	N/A

**Oligonucleotides**		

Nested PCR Primers (see Table S2)	This manuscript and McCoy et al., 2016	N/A

**Software and Algorithms**		

Prism v7	GraphPad	RRID:SCR_002798
FlowJo	FlowJo	RRID:SCR_008520
UCSF Chimera	Pettersen et al., 2004	RRID:SCR_004097
Appion database	Lander et al., 2009	N/A
Leginon	Suloway et al., 2005	RRID:SCR_016731
DoG Picker	Voss et al., 2009	RRID:SCR_016655
Relion	Scheres, 2012	RRID:SCR_016274
PyMOL	Schrödinger	RRID:SCR_000305
SWISS-MODEL	Arnold et al., 2006	RRID:SCR_013032
Phenix	Adams et al., 2010	RRID:SCR_014224
Coot	Emsley and Cowtan, 2004	RRID:SCR_014222
EMRinger	Barad et al., 2015	fraserlab.com/2015/02/18/EMringer
MolProbity	Williams et al., 2018	RRID:SCR_014226
Byologic v2.3	ProteinMetrics	https://www.proteinmetrics.com/products/byonic/
Byonic v2.7	ProteinMetrics	https://www.proteinmetrics.com/products/byonic/
Rosetta	Conway et al., 2014	RRID:SCR_015701
GCTF v1.06	Zhang 2016	RRID:SCR_016500

**Other**		

Streptavidin (SA) Biosensors	ForteBio	Cat#18-5020
Anti-human Fc capture (AHC) Biosensors	ForteBio	Cat#18-5063
Anti-His (HIS2) Biosensors	ForteBio	Cat#18-5114

### Lead Contact and Materials Availability

Further information and requests for resources and reagents should be directed to and will be fulfilled by the Lead Contact, Richard T. Wyatt (wyatt@scripps.edu). Plasmids generated in this study are available upon request to the Lead Contact.

### Experimental Model and Subject Details

#### Rabbits

The rabbit immunogenicity study was carried out under subcontract at Covance (Denver, PA), a site approved by the Association for Assessment and Accreditation of Laboratory Animal Care (AAALAC). The Covance Institutional Animal Care and Use Committee (IACUC) approved the study protocol (#0122-16), which was designed and conducted in strict accordance with the recommendations of the NIH *Guide for the Care and Use of Laboratory Animals*, the Animal Welfare Act and under the principles of the 3Rs. All efforts were made to minimize discomfort related to the inoculations and blood collection. Female New Zealand white rabbits (Covance Research Products), approximately 7 mo old at the start of the study, were singly housed in stainless steel caging in Covance’s barrier facility for the duration of the study.

#### Cell Lines

TZM-bl cells (human female HeLa-derived cancer cell line; NIH AIDS reagent program) and 293T (human female embryonic kidney; ATCC) cells were cultured in high glucose Dulbecco’s Modified Eagle Medium (DMEM, GIBCO) containing 1X Penicillin-Streptomycin (GIBCO), 2 mM L-Glutamine (GIBCO), and 10% heat-inactivated FB Essence (Seradigm), at 37°C and 5% CO_2_. FreeStyle 293F (human embryonic kidney; GIBCO) cells were cultured in serum-free FreeStyle expression medium (Life Technologies) in suspension at 37°C and 8% CO_2_.

### Method Details

#### Design, expression and purification of HIV Env constructs

The design of the WT trimer immunogens for HIV Env strains (16055, JRFL, BG505, 001428, ZM197M) in the NFL TD CC+ platform as well as the 16055 NFL TD CC+ glycan mutants have been previously described (Dubrovskaya et al., 2017, Guenaga et al., 2017). Similarly, we generated the N glycan mutants: JRFL NFL TD CC+ ΔGly1 (N276Q) and ΔGly2 (N276Q/N463Q) as well as BG505 NFL CC+ ΔGly1 (N276Q) and ΔGly2 (N276Q/N462Q). For the sera mapping assays, we produced 16055 gp120 TriMut and corresponding CD4bs knockout (D368R/M474A) as previously described (Feng et al., 2016, Martinez-Murillo et al., 2017). TriMut refers to three mutations (I423M, N425K, and G431E) that inhibit binding to CD4 on the TZM-bl target cells, but does not affect recognition by CD4bs-directed mAbs, so the proteins can be present in the neutralization assay (Feng et al., 2012). The CD4bs knockout mutations (D368R/M474A) eliminates recognition by CD4bs-directed mAbs. For the polyclonal sera mapping by EM, we generated SC422 NFL TD CC+, 45_01dG5 NFL TD 2CC+ and 45_01dH5 TD 2CC+ trimers based on the NFL TD CC+ platform with an additional disulfide (501C-663C) (Guenaga et al., 2017, Yang et al., 2018). Changes in the coding sequence to generate the described Env mutants were introduced by site-directed mutagenesis using a QuikChange Lightning Multi Site-Directed Mutagenesis Kit (Agilent Technologies). All modifications were confirmed by DNA sequencing. Trimers contained a C-terminal His-tag for coupling to Ni-NTA liposomes.

The Env proteins were transiently expressed as soluble glycoproteins in 293F cells using the FreeStyle 293 Expression System (Life Technologies, Thermo Fisher) per manufacturer’s protocol. Briefly, 300 μg purified plasmid DNA was complexed with 750 μl 293fectin (Life Technologies, Thermo Fisher) in Opti-MEM media at RT for 30 min and then added to 1 L of 293F cells at 1.2 × 10^6^ cells/ml. Cell culture supernatants were harvested at day 5 post-transfection, and the Env-derived glycoproteins were purified by affinity chromatography using a *Galanthus nivalis* lectin-agarose column (Vector Laboratories). Eluted glycoproteins were further purified by size-exclusion chromatography (SEC) using a HiLoad Superdex 200 16/60 column or Superdex 200 Increase 10/300 GL column (GE Healthcare). The trimer peak fractions were pooled and subjected to negative selection over a F105 or GE136 affinity column to remove any residual monomer/dimer or open trimers as needed (Guenaga et al., 2015b).

#### Site-specific glycan analysis

Site-specific glycan analysis of the trimers was performed as previously described (Behrens et al., 2016). Approximately 100 μg of each trimer was denatured in 50 mM Tris/HCl, 6 M Urea, pH 8.0, reduced in 5 mM dithiothreitol (DTT) for 1 hr at RT, followed by alkylation in 20 mM iodacetamide (IAA) for 1 hr. Residual IAA was neutralized for 1 hr in 20 mM DTT. The trimers were buffer exchanged into 50 mM Tris/HCl, pH 8.0 using Vivaspin columns and then digested with either trypsin or chymotrypsin (Mass Spectrometry Grade, Promega) at a ratio of 1:30 (w/w) overnight at 37°C. The reaction mixture was dried in a SpeedVac concentrator, and glycopeptides/peptides were extracted using C18 ZipTips (MerckMillipore) following the manufacturer’s instructions. Glycopeptides/peptides were resuspended in 5 μl 0.1% formic acid and a 1 μl aliquot was analyzed by in-line, liquid chromatography-electrospray ionisation mass spectrometry (LC-ESI MS), with an Easy-nLC 1200 system coupled to a Fusion mass spectrometer (Thermo Fisher Scientific), using higher energy collisional dissociation (HCD) fragmentation. HCD energy was set to 50%. Glycopeptide fragmentation data was extracted from the raw file using Byonic (Version 2.7) and Byologic software (Version 2.3; Protein Metrics) and data were manually validated as described (Behrens et al., 2018). To assign remaining sites as oligomannose/hybrid-type, complex-type or unoccupied, the remaining glycopeptides/peptides were digested with Endoglycosidase H for 4 hr at 37°C, dried, then digested with Peptide N-Glycosidase F (PNGase F) in O^18^-labeled water for 1 hr, as described in Cao et al. (2017). Digested peptides were dried, extracted and analyzed as above, but with a lower HCD energy of 27%.

Models were generated using SWISS-MODEL (Arnold et al., 2006), based on homology to structures: PDB: 5FUU (JRFL gp120), 5FYK (JRFL gp41), 5FYJ (ZM197 gp41), and 5UM8 (ZM197 gp120, 16055 and 001428). Glycans were modeled on using Coot, assigning the predominant glycan type at each site according to the intact glycopeptide site-specific analysis. Where only O^18^ site-specific data was available, Man_5_GlcNAc_2_ was modeled. BG505 was modeled according to Behrens et al. (2016).

#### Thermostability of soluble Env trimers

Thermal stability of the soluble Env trimers was evaluated using a MicroCal VP-Capillary differential scanning calorimetry (DSC) instrument (Malvern) as previously described (Dubrovskaya et al., 2017, Guenaga et al., 2015b). Sample concentration was adjusted to 0.125 mg/ml. Scans were collected at a rate of 1 K/min under 3.0 atmospheres of pressure. DSC data were analyzed after buffer correction, normalization, and baseline subtraction using CpCalc software provided by the manufacturer.

#### Bio-layer light interferometry (BLI) binding analysis

An Octet RED96 system (ForteBio) was used for antigenicity assessment of the various trimers by BLI analysis. Trimers were loaded onto anti-HIS biosensors (HIS2, ForteBio) at 10 μg/ml and Abs (200 nM) were used as the analyte for binding analysis. The assays were performed at 30°C at a shaking speed of 1,000 rpm. Data Analysis 7.0 evaluation software (ForteBio) was used to assess the response curves.

#### Enzyme-linked immunosorbent assay (ELISA)

His-capture ELISA was performed as previously described (Dubrovskaya et al., 2017) to evaluate trimer antigenicity and to measure elicited trimer-specific, IgG binding titers in the rabbit sera. MaxiSorp plates (Nunc, Thermo Fisher) were coated overnight at 4°C with 1.5 μg/ml of a mouse anti-His tag mAb (MAB050, clone AD1.1.10; R&D Systems) in PBS, pH 7.4. Plates were blocked with 2% BSA in PBS, pH 7.4. Following incubation with soluble His-tagged Env trimers (3 μg/ml in PBS, pH 7.4), serially diluted mAbs or sera were added for 1 hr, after which secondary Abs (peroxidase-conjugated goat anti-human IgG or anti-rabbit IgG) were incubated for another hr. Plates were washed with PBS containing 0.1% Tween-20 between each incubation step and developed with 3,3′,5,5;-tetramethylbenzidine chromogenic substrate solution (Life Technologies). Reactions were stopped with sulfuric acid and the plates were read at A450.

For direct-coat ELISA, proteins were added directly to the wells at 2 μg/ml and analyzed for Ab binding as described above. A non-HIV, C-terminal His-tagged protein (i.e., HA-His) was used to detect anti-His responses as described above.

#### Trimer-liposome conjugation

Preparation of the liposomes and protein conjugation were performed as previously described (Ingale et al., 2016). In brief, liposomes were prepared using a mixture of DSPC (1,2-distearoyl-sn-glycero-3-phosphocholine), cholesterol, and DGS-NTA(Ni^2+^) ((1,2-dioleoyl-*sn*-glycero-3-[(*N*-(5-amino-1-carboxypentyl) iminodiacetic acid)succinyl] (nitrilotriacetic acid nickel salt)) in a molar ratio of 60:36:4, respectively. The components were dissolved in chloroform, mixed and desicated overnight under vacuum. The formed lipid film was hydrated in PBS for 2 hr at 37°C, with constant shaking followed by sonication. The liposomes were extruded by sequentially passing through 1.0, 0.8, 0.2, and 0.1 μm membrane filters (Whatman Nuclepore Track-Etch membranes). To conjugate the His-tagged trimers, 900 μg total protein was incubated with 300 μl liposomes. The trimer-liposomes were separated from excess free protein using a Superdex 200 size-exclusion column. Trimer-liposome fractions were pooled and quantitated using a standard curve generated by soluble trimer using the Advanced Protein Assay Reagent (Cytoskeleton). Samples were stored at 4°C and checked by EM negative stain analysis and BLI for antigenicity prior to each immunization as previously described (Bale et al., 2017).

#### Animal immunization, sampling, and sample preparation

New Zealand white rabbits (6 per group) were immunized subcutaneuosly with bilateral inoculations over the hips (100 μl per side), consisting of 30 μg trimer-liposomes formulated with 75 U of ISOCMATRIX adjuvant (CSL) [or the similar ISCOMs-class saponin adjuvant (Pauthner et al., 2019; gift from Darrell Irvine) for the last two immunizations] and sterile PBS, pH 7.4. The female rabbits, which were approximately 7 months old and weighed ∼3 kg at the beginning of the study, were randomly assigned to the different groups. Blood samples were collected pre and 2 wks post immunization. Lymph nodes (popliteal and inguinal) and spleens were taken at the end of the study.

Peripheral blood mononuclear cells (PBMCs) were isolated from plasma using Ficoll-Paque PLUS density gradient media (GE Healthcare), washed extensively in PBS treated with red blood cell lysis buffer (ACK lysis buffer), and frozen in Bambanker or FBS freezing media (90% heat-inactivated FBS/10% dimethyl sulfoxide). Spleens or lymph nodes (popliteal and inguinal combined) were collected in sterile chilled R10 media (RPMI 1640, 10% heat inactivated FBS, 1% Pen-Strep). Lymph nodes were mashed, washed extensively with chilled R10 media, and filtered through 70 μm cell strainers before counting and freezing in Bambanker or FBS freezing media. Spleen tissues were subjected to an additional incubation with ACK lysis buffer to remove red blood cells, extensively washed and filtered before the splenocytes were counted and frozen.

#### HIV-1 neutralization assays

Standard TZM-bl-based neutralization assays were performed as previously described (Li et al., 2005) using various HIV-1 Env pseudoviruses. Site-directed mutagenesis of the Env coding sequence was used to generate the different Env mutants. Pseudoviruses were pre-incubated for 1 hr with serial dilutions of serum samples, purified total serum IgGs, or mAbs before addition to the TZM-bl cells. For the time course evaluation, mAbs were pre-incubated with virus for 1, 6, 12, or 24 hr. Neutralization dose-response curves were fit by non-linear regression using a 5-parameter hill slope equation. Neutralization capacity was expressed as the serum dilution factor (ID_50_) or as the IgG/mAb concentration (IC_50_, μg/ml) sufficient to inhibit virus infection by 50%, as measured by relative luciferase units. Neutralization assays were performed at least twice.

For the solid-phase adsorption assay, Env trimers were coupled to *Galanthus nivalis* lectin-agarose beads (350 μg trimer to 200 μl bead slurry (50% in PBS) overnight at 4°C on a rotating mixer. Beads were washed extensively in PBS to remove unbound trimer. Purified rabbit serum IgG, mAb controls, or equivalent volume of PBS were incubated with the coupled beads at RT for 1 h with regular gentle pipetting to mix the beads with the sample. After centrifuging at 2500 x g for 1 min to pellet the beads, the supernatant was carefully removed to be used in the neutralization assay.

To map neutralization to the CD4bs, a differential inhibition of neutralization assay was performed using isogenic TriMut probes, which contain I423M/N425K/G431E mutations to inhibit CD4 binding and thereby not interfere with the CD4-dependent HIV-1 entry assay, as described previously (Feng et al., 2012). Serial dilutions of 16055 gp120 TriMut proteins, WT or containing D368R/D474A mutations to inhibit binding to CD4bs-directed mAbs, or cell culture medium (negative control) were pre-incubated with the predetermined IC_80_ concentration of purified serum IgG for 1 hr at 37°C prior to incubation with pseudovirus.

#### Env-specific B cell sorting by flow cytometry

Env-specific B cells were single cell sorted from cryopreserved PBMCs, lymph nodes (popliteal or inguinal) or splenocytes from rabbit C3 collected two weeks after the sixth immunization. Single, live cells (as determined by a Live/Dead cell stain [Molecular Probes, Invitrogen] or DAPI) were sorted by gating IgG+ and Env+ cells into 96-well PCR plates containing cell lysis buffer. Plates were sealed, immediately frozen on dry ice and stored at −80°C. Env probes (monomeric gp120 or stabilized NFL trimers) containing Avitags were biotinylated using BirA (Avidity) and conjugated with a streptavidin flurochrome (Molecular Probes). Initial sorting strategy using differential probes (16055 gp120+ and 16055 gp120 D368R-) to gate for CD4bs-specific cells yielded few single positive B cells. Selected heterologous probes were then used to select for cross-binding IgG+ B cells. To enrich for potentially (tier 2) cross-neutralizing B cells, 16055, used in the immunogen series, was paired with heterologous Envs (e.g., SC422 and 1086) that were not included as an immunogen but are neutralized by C3 sera/IgG. Monomer-trimer pairs were used to select for cross-binding gp120 epitopes (e.g., the CD4bs) while excluding potentially non-neutralizing CD4bs or base responses. Trimer-trimer pairs were used to sort for cross-binding and potentially trimer-specific responses. In total, approximately 800 B cells were sorted using the selected probe sets (see Table S2). All fluorescently labeled Abs and probes were carefully titrated to enhance specificity.

#### Single B cell RT-PCR and antibody expression

RNA from the sorted Env-specific single B cells from rabbit C3 were reversed transcribed and used for IgG V(D)J sequence amplification based on protocols as described previously (McCoy et al., 2016, Sundling et al., 2012) with some modification. Briefly, the RNA was reverse transcribed to cDNA using random hexamers (Gene Link), dNTPs (Sigma), RNaseOUT (Life Technologies) and SuperScript III or IV reverse transcriptase (Life Technologies). Heavy and light chain V(D)J segments were amplified by nested PCR using HotStar Taq Plus (QIAGEN) with 5′ leader sequence-specific and 3′ IgG constant region-specific primers. Along with the previously published primers (McCoy et al., 2016), additional primers were designed for optimization and to aid coverage (Table S3). PCR products were purified and sequenced. Heavy and light chain variable regions were PCR amplified with primers containing homology arms specific for the heavy/light chain expression vector (McCoy et al., 2016), and PCR products were ligated into the expression vector using NEBuilder high fidelity DNA assembly mix (NEB) as previously described (McCoy et al., 2016). For Fab expression, an alternative HC expression vector was generated to incorporate a C-terminal 6x-His-tag and stop codon in the hinge region.

Rabbit mAbs and Fabs were transiently expressed using FreeStyle 293 Expression System (Invitrogen) as described above. mAbs were purified using Protein A Sepharose Fast Flow (GE Healthcare) resin. His-tag Fabs were affinity purified with Nickel resin followed by SEC. The purified mAbs and Fabs were analyzed by SDS-PAGE to confirm purity and subsequently tested for Env-specific binding and neutralization. Of the ninety-nine mAbs expressed, twenty-one showed neutralizing activity (see Tables S2 and S4).

#### Env trimer dissociation by BN-PAGE

Virus samples were incubated at 37°C with Fab for 0-24 hr. The Env/Fab complexes were subsequently solubilized from the membrane using 1% *n*-dodecyl-β-d-maltoside (DDM). Blue native PAGE (BN-PAGE) using the Native PAGE bis-Tris gel system (Invitrogen) as previously described (Rujas et al., 2018). Samples were run on a 3%–8% gradient Tris-acetate gel (Invitrogen) at 150 V for 3 hr at 4°C. Proteins in the gel were transferred to a PVDF membrane, and membranes were blotted overnight using a cocktail of gp120-specific mAbs (2 μg/ml ea of b12, 2G12, and 447-52D) and gp41-specific mAbs (1 μg/ml ea of 10E8, 2F5, and 7B2) combined at 4°C. Ab binding was detected using a HRP-conjugated goat anti-human Fc Ab (Jackson ImmunoResearch), and peroxidase activity was assayed using ECL Plus Western Blotting Substrate (Pierce). BN-PAGE analysis was performed twice independently.

Soluble NFL Env trimers (10 μg) were pre-incubated with or without Fab (20 μg) at 37°C for 0 h or 22 hr and analyzed by BN-PAGE. Samples were run on a 3%–8% Bis-Tris gradient gel and stained with Coomasie Blue.

#### Flow cytometry analysis of cell-surface HIV-1 Envs

FACS staining was performed as previously described (Pancera and Wyatt, 2005). Forty-eight hr following Env transfection, 293T cells were harvested, washed in FACS buffer (PBS, 5% FBS), and stained with a panel of serially diluted mAbs. After extensive washing in FACS buffer, the cells were incubated with phycoerythrin (PE)-conjugated anti-human or anti-rabbit secondary Abs (Jackson ImmunoResearch) at a 1:200 dilution, followed by extensive washing to remove unbound secondary antibody. The PE-stained cells were analyzed by flow cytometry on a NovoCyte instrument (ACEA Bioscience).

#### Negative-stain electron microscopy (nsEM)

Complexes were formed by incubating a 6-10x molar excess of Fab to trimer: E70 Fab with BG505 NFL CC+; 1C2 Fab with 16055 NFL TD CC+ or 2CC+. The complexes were purified by size exclusion chromatography and 3 μl of either complex (at 0.03 mg/ml in 1X TBS pH 7.4) were deposited on carbon-coated copper mesh grids (Electron Microscopy Sciences), which had been previously plasma cleaned with a Gatan Solarus Advanced Plasma Cleaning System for 20 s with an Argon/Oxygen mix. Grids were negatively stained with 2% (w/v) uranyl formate for 50 s and loaded into an FEI Tecnai Spirit (120 keV) electron microscope equipped with a TVIPS TemCam F416R camera for data collection, which was facilitated by the Leginon software suite (Suloway et al., 2005). Micrographs were stored in the Appion database (Lander et al., 2009). Particles were picked with Dogpicker (Voss et al., 2009) and were stacked with a box size of 160 pixels. The particle stack was exported to Relion (Scheres, 2012), where 2D and 3D classifications, and 3D refinements were performed. A 30 Å low-pass filtered trimer (PDB 4ZMJ) was used as the initial model and C3 symmetry was applied to the final refinements. A total of 20,865 particles were used for the final reconstruction of BG505 NFL CC+ in complex with E70, and 6,000 particles for 16055 NFL TD 2CC+ in complex with 1C2. Maps were evaluated using UCSF Chimera (Pettersen et al., 2004). Resolution estimates for both maps are ∼21 Å based on an FSC cutoff of 0.5.

For polyclonal sample analysis, similar methods (dilutions, grid preparation, staining, data collection/processing) were used as above. In addition to the FEI Tecnai Spirit/TVIPS TemCam F416R combination above (120 keV; 52,000x magnification; 2.05 Å/pix), data were also collected using a Thermo Fisher Talos F200C equipped with a Thermo Fisher Ceta 16M camera (200 keV; 73,000x magnification, 1.98 Å/pix), or an FEI TF20 equipped with an FEI Eagle 4K camera (200 keV; 62,000x magnification; 1.79 Å/pix). Classifications and 3D reconstructions were performed using Relion 3.0 (Scheres, 2012).

#### Cryo-electron microscopy sample preparation

Complexes were formed in a similar manner as described above for nsEM. CryoEM grids were prepared using a Thermo Fisher Vitrobot Mark IV set to 100% humidity, 10°C, and a 10 s wait time. BG505 NFL CC+ in complex with E70 Fab was briefly incubated with lauryl maltose neopentyl glycol (LMNG) at final concentrations of ∼5 mg/mL protein complex and 0.005 mM detergent. 16055 NFL TD 2CC+ in complex with 1C2 Fab was briefly incubated with n-Dodecyl-β-D-Maltoside (DDM) at final concentrations of ∼5 mg/mL protein complex and 0.06 mM detergent. 3 μL of either complex was deposited on Ar/O_2_ plasma cleaned C-Flat 2/2-4C copper mesh grids. The complex was blotted for 5 s before plunge freezing in liquid ethane.

#### Cryo-electron microscopy data collection, processing and model refinement

Statistics for cryoEM data collection, processing and model refinement are summarized in Table S5. Briefly, the BG505 NFL CC+ / E70 Fab complex was collected using a Thermo Fisher Titan Krios (300 keV) and a Thermo Fisher Gatan K2 Summit (4K x 4K) camera. A total of 870 movie micrographs were collected at 29,000x magnification with a defocus range of −0.5 to −2 μm, and a pixel size of 1.03 Å. The total dose was 57 e^-^/ Å^2^ which was fractionated over 48 frames, each receiving 5.04 e^-^/pix/sec for 250 ms. For the 16055 NFL TD 2CC+ / 1C2 Fab complex, data were collected on a Thermo Fisher Talos Arctica (200 keV) paired with a Gatan K2 Summit (4K x 4K) camera. A total of 1,690 movie micrographs were collected at 36,000x magnification with a pixel size of 1.15 Å. The total dose was 48 e^-^/ Å^2^ which was fractionated over 50 frames, each receiving 5.10 e^-^/pix/sec for 250 ms. Micrographs were collected using the Leginon software suite (Suloway et al., 2005) and stored in the Appion database (Lander et al., 2009).

CTF correction was performed using GCTF v1.06 (Zhang, 2016) and image processing was done using Relion 3.0 (Scheres, 2012). Following several rounds of 2D and 3D classification, C3 symmetry was imposed in the final reconstructions and the estimated resolutions based on an FSC 0.143 cutoff are ∼3.6 Å (E70:BG505 NFL CC+ with 49,635 total particles) and ∼3.9 Å (1C2:16055 NFL TD 2CC+ with 23,702 total particles).

Models were built by generating homology models using the 1C2 Fab crystal structure in this study, and either PDB 6B0N (X-ray structure of BG505 NFL) or PDB 5UM8 (X-ray structure of 16055 NFL TD CC+). We modeled the E70 F_v_ *de novo* into the cryoEM density. All models were refined iteratively by manual manipulation in Coot (Emsley and Cowtan, 2004) and automated relaxed refinement using Rosetta (Conway et al., 2014). Final statistics were assessed using MolProbity (Williams et al., 2018) and EMRinger (Barad et al., 2015), and are summarized in Table S5.

#### Crystallography, data collection, refinement and model building

Purified 1C2 Fab in HBS buffer (5 mM HEPES, 150 mM NaCl) was concentrated down to an optical density of 18 and screened against the Hampton Crystal HT, ProPlex HT-96, and Wizard Precipitant Synergy #2 crystallization screens. The NT8 robotic system was used to set initial sitting drop crystallization trials. Following initial hits, crystallization conditions were optimized using hanging drop vapor diffusion. Crystals for the 1C2 Fab appeared within a few days. The final condition of 1C2 crystals used for data collection was 0.1M Imidazole 6.5, 16% PEG 3350, 10% MPD, and 0.2M Lithium Sulfate. Crystals used for data collection were taken directly from the drop and flash frozen in liquid nitrogen. Diffraction data for 1C2 was collected at APS beamline ID22. Diffraction data was processed using HKL2000, and 1C2 molecular replacement solutions was found in Phenix searching with PDB 4JO3. Following MR, COOT was used for additional model building and Phenix (Adams et al., 2010) for refinement. Data collection and refinement statistics are summarized in Table S6.

### Quantification and Statistical Analysis

All binding and neutralization assays were conducted with at least duplicate measurements.

### Data and Code Availability

Data generated or analyzed during this study are included in this published article and supplemental information. CryoEM and EM reconstructions have been deposited in the Electron Microscopy Data Bank (EMD: 20259, EMD: 20260, EMD: 20273, EMD: 20274, EMD: 20279, EMD: 20280, EMD: 20342, EMD: 20345, EMD: 20346, EMD: 20347) and in the Protein Data Bank (PDB: 6P62 and PDB: 6P65). Atomic coordinates and structure factors of the reported crystal structure have been deposited in the Protein Data Bank (PDB: 6PEH).

## Supplemental Information

Document S1. Figures S1–S7 and Tables S1–S6

Document S2. Article plus Supplemental Information
