# Supplementary material for: Vaccination with Glycan-Modified HIV NFL Envelope Trimer-Liposomes Elicits Broadly Neutralizing Antibodies to Multiple Sites of Vulnerability
Source: Immunity. 2019 Nov 19;51(5):915–929.e7. doi: 10.1016/j.immuni.2019.10.008 (PMC6891888; doi:10.1016/j.immuni.2019.10.008)
Supplement: Document S1. Figures S1–S7 and Tables S1–S6 [file mmc1.pdf]

Immunity, Volume *51*

Supplemental Information

Vaccination with Glycan-Modified HIV NFL Envelope Trimer-Liposomes Elicits Broadly Neutralizing Antibodies to Multiple Sites of Vulnerability

Viktoriya Dubrovskaya, Karen Tran, Gabriel Ozorowski, Javier Guenaga, Richard Wilson, Shridhar Bale, Christopher A. Cottrell, Hannah L. Turner, Gemma Seabright, Sijy O'Dell, Jonathan L. Torres, Lifei Yang, Yu Feng, Daniel P. Leaman, Néstor Vázquez Bernat, Tyler Liban, Mark Louder, Krisha McKee, Robert T. Bailer, Arlette Movsesyan, Nicole A. Doria-Rose, Marie Pancera, Gunilla B. Karlsson Hedestam, Michael B. Zwick, Max Crispin, John R. Mascola, Andrew B. Ward, and Richard T. Wyatt

Figure S1

A

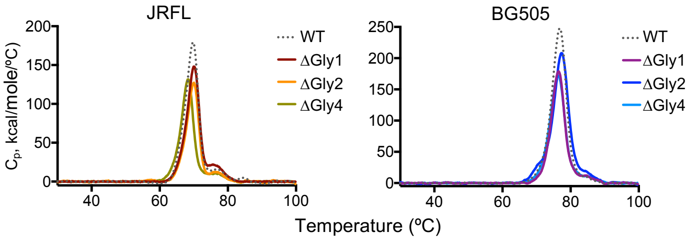

Env	WT	ΔGly1	ΔGly2	ΔGly4
16055 NFL TD CC+	71.6ºC	71.4ºC	71.5ºC	71.2ºC
JRFL NFL TD CC+	69.8ºC	70.1ºC	70.0ºC	68.4ºC
BG505 NFL CC+	76.9ºC	76.4ºC	77.4ºC	76.2ºC
001428 NFL TD CC+	68.0ºC	N/A	N/A	N/A
ZM197M NFL TD CC+	67.0ºC	N/A	N/A	N/A

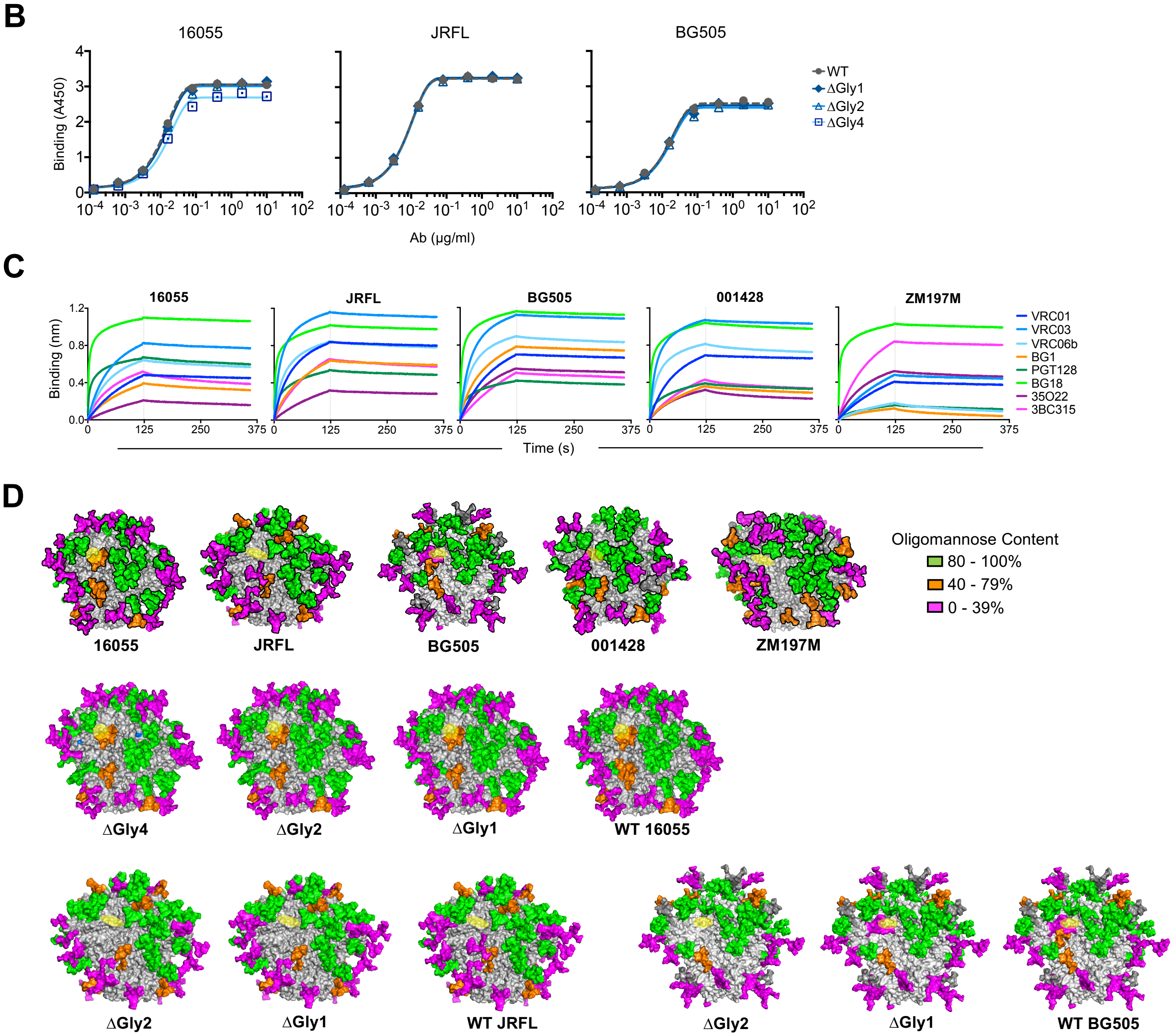

Figure S1. Biophysical characterization of immunogens, related to Figure 1 and Table S1. (A) Thermostability of glycan-deleted trimers compared to parental (WT), as determined by differential scanning calorimetry. Tm values are summarized in the table to the right. (B) Antigenicity of the glycan-deleted trimers compared to parental (WT), as determined by His-capture ELISA. (C) Relative binding affinity of the NFL trimers to a panel of CD4bs-directed mAbs, as determined by BLI. GE136, which is non-neutralizing and does not bind to well-folded trimers, was used as a negative control. (D) Site-specific glycan analysis of all immunogens. Glycans are colored by oligomannose content. CD4bs is marked in yellow for reference.

Figure S2

A

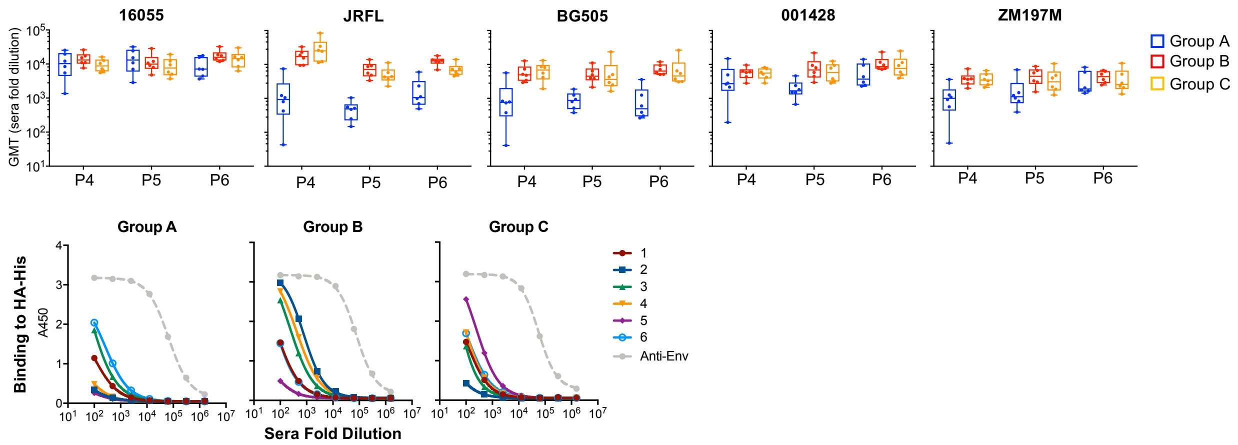

B

Virus	A: WT Homologous ID# 1	2	3	4	5	6	B: ΔGly Prime: Seq Het Boost 1	2	3	4	5	6	C: ΔGly Prime: Seq Cocktail Het Boost 1	2	3	4	5	6
16055	210		883	1043	22	337	37		183			52		26	923		566	21
JRFL							70			596	51		17		40	13		
BG505	10									16			16		1778	12	12	11
001428										20		39		23	1209			
ZM197	12						15			39	14	27		23	1906	237	44	
SIVmac239																		

ID50 (sera dilution): <10; 10-100; 100-1000; >1000

C

Post 5

Virus	Clade	WT — Grp A: WT Homologous ID# 1	2	3	4	5	6	ΔGlycan Priming:Heterologous Boost — Grp B: ΔGly Prime: Seq Het Boost 1	2	3	4	5	6	Grp C: ΔGly Prime: Seq Het Cocktail Boost 1	2	3	4	5	6
RW020	A												1808			12			
3301.V1	AC	858											147			347			
45_01dG5	B										451		237	1362		0.27			
QH0515	B	929																	
RHPA	B															2.62			
SC422	B												50			50			
TRO.11	B												301			13			
BJOX002000	BC												191			139			
CH119	BC												166			53			
IAVIC22	C										322	315	60			51			
ZM249	C										821			1883		336			1698
NKU3006	D															15			
SIVmac251																			

IgG IC50 (μg/ml): >2000; 1000-2000; 100-1000; 10-100; 1-10; <1

D

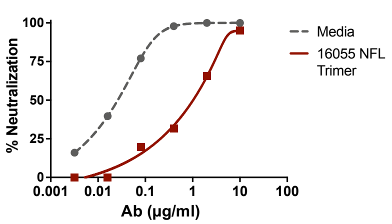

E

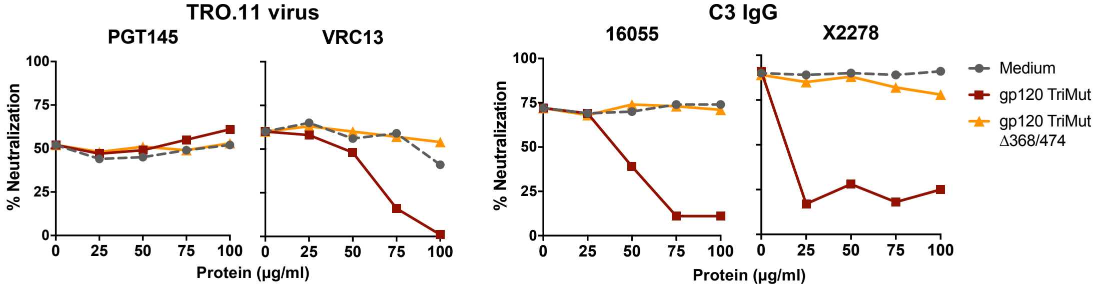

Figure S2. Characterization of vaccine-elicited neutralizing responses reveals cross-neutralization in multiple subjects, related to Figure 2. (A) Geometric mean IgG titers (GMT, ±SD; *n* = 6) are shown above for Post 4 to Post 6 immunizations, as measured by His-capture ELISA to the different WT immunogens. Below, anti-His binding responses directed against HA-His protein from Post 6 sera, as measured by ELISA with animal numbers from each group as indicated. The sera were also assessed against 16055 NFL trimer with the average response for each group shown as a gray dashed line for reference. (B) Post 6 sera neutralizing titers (ID_{50}, sera-fold dilution at which 50% neutralization is achieved) are indicated against the panel of viruses used to derive the sequential Env trimer immunogens. (C) Cross-neutralization of purified IgG (IC_{50}, μg/ml) from the Post 5 time point against a panel of representative (tier 2) clinical HIV-1 isolates across different clades. (D) The trimer-specific PGT145 bNAb served as a positive control for the solid-phase adsorption assay using a 16055 NFL TD CC+ trimer affinity column to deplete Env binding Abs prior to neutralization assessment against virus TRO.11. A PBS-immersed (media) blank lectin column was used as a negative control. (E) Differential adsorption of virus entry. Left, PGT145 or VRC13 (CD4bs-directed bNAb) were pre-incubated at a fixed concentration with culture medium (no inhibitor) or titrating amounts of WT or the CD4bs knockout (368/474) 16055 gp120 TriMut proteins prior to assessing neutralization against virus TRO.11 as negative and positive controls. Right, purified IgG from C3 anti-sera (Post 5) was pre-incubated at a fixed concentration with medium or titrating amounts of the TriMut proteins prior to assessing neutralization against viruses 16055 and X2278. All assays were performed at least twice with representative data shown.

Figure S3

A

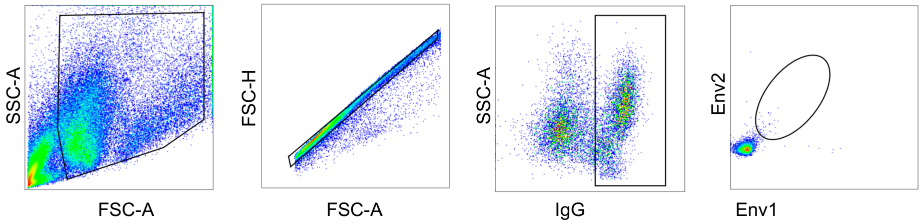

B

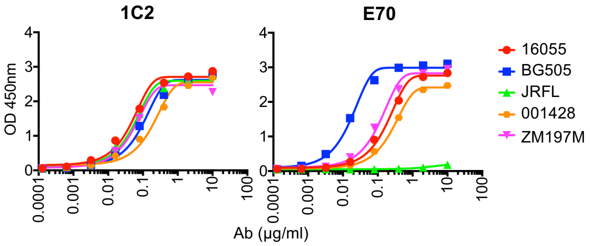

C

mAb	Sorted From	Probes	Chain	V-GENE	J-GENE	D-GENE	CDR3-IMGT	CDR3, aa	SHM, %*
**E70**	Splenocytes	16055 gp120 SC422 NFL TD CC+	HC	IGHV1S40*01 F	IGHJA*01 F	IGHD3-1*01	RFAGYRYSVWSYPDL	15	8
			KC	IGKV1S36*01 [F]	IGKJ1-2*01 F		QSAYNGDGDNA	11	8
**1C2**	Lymph nodes	16055 NFL TD CC+ 1086c NFL TD CC+	HC	IGHV1S40*01 F	IGHJ4*01 F	IGHD8-1*01	ARGAVTYDGLGGAYLKHFNL	20	5
			KC	IGKV1S47*01 [F]	IGKJ1-4*03		LYGYISSDRID	11	6

D

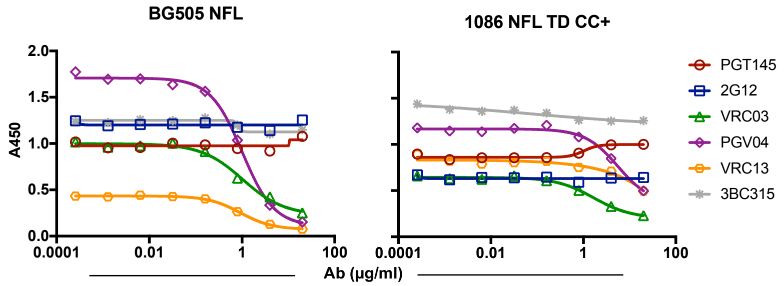

E

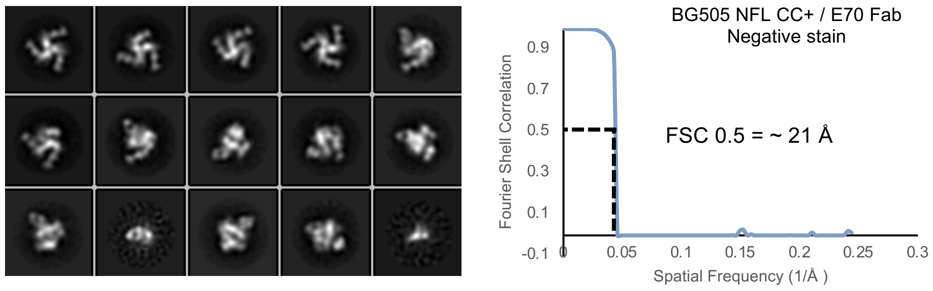

F

N-Glycan Dependence

	16055	BG505	1086.B2
WT	0.96	0.05	0.03
N234A	>50	>50	>50
N234Q	>50	>50	ND
(T/S)236A	>50	>50	ND
N276A	0.04	0.03	ND
N360A	0.35	ND	ND
N463A	0.99	ND	ND
295N	1.95	ND	ND
339N	2.31	ND	ND
355N	0.74	ND	ND

N234 Restoration

	ZM249M	JRFL
WT	>50	>50
K236T	2.33	>50

N276 Deletion

	001428	IAVIC22	92TH021
WT	>50	>50	2.13
N276A	0.012	0.46	0.018

Figure S3. Isolation and characterization of two mAbs from rabbit C3 that can cross-neutralize HIV clinical isolates, related to Figures 2-3 and Table S2-S4. (A) FACs plots showing the gating strategy used for sorting single, dual Env+ B cells from rabbit C3 samples collected two weeks after the sixth immunization. (B) Binding of isolated cross-neutralizing rabbit mAbs, 1C2 and E70, against the WT immunogens used in the study by His-capture ELISA. (C) Summary Table of E70 and 1C2 features based on currently available information in the IMGT database. *, max SHM% calculated based on putative gene assignments. (D) Cross-competition ELISA of E70 against a panel of representative bNAbs targeting different Env epitopes: PGT145 (V2), 2G12 (N332 glycan supersite), CD4bs (VRC03, PGV04, VRC13), 3BC315 (gp120:gp41 interface). (E) EM 2D class averages of E70 Fab bound to BG505 NFL CC+ trimer with the Fourier shell correlation graph shown. (F) Neutralization assessment of E70 against a panel of N-glycan mutants (deleted or restored) to determine N-glycan sensitivity. IC_{50} (µg/ml) values shown.

Figure S4

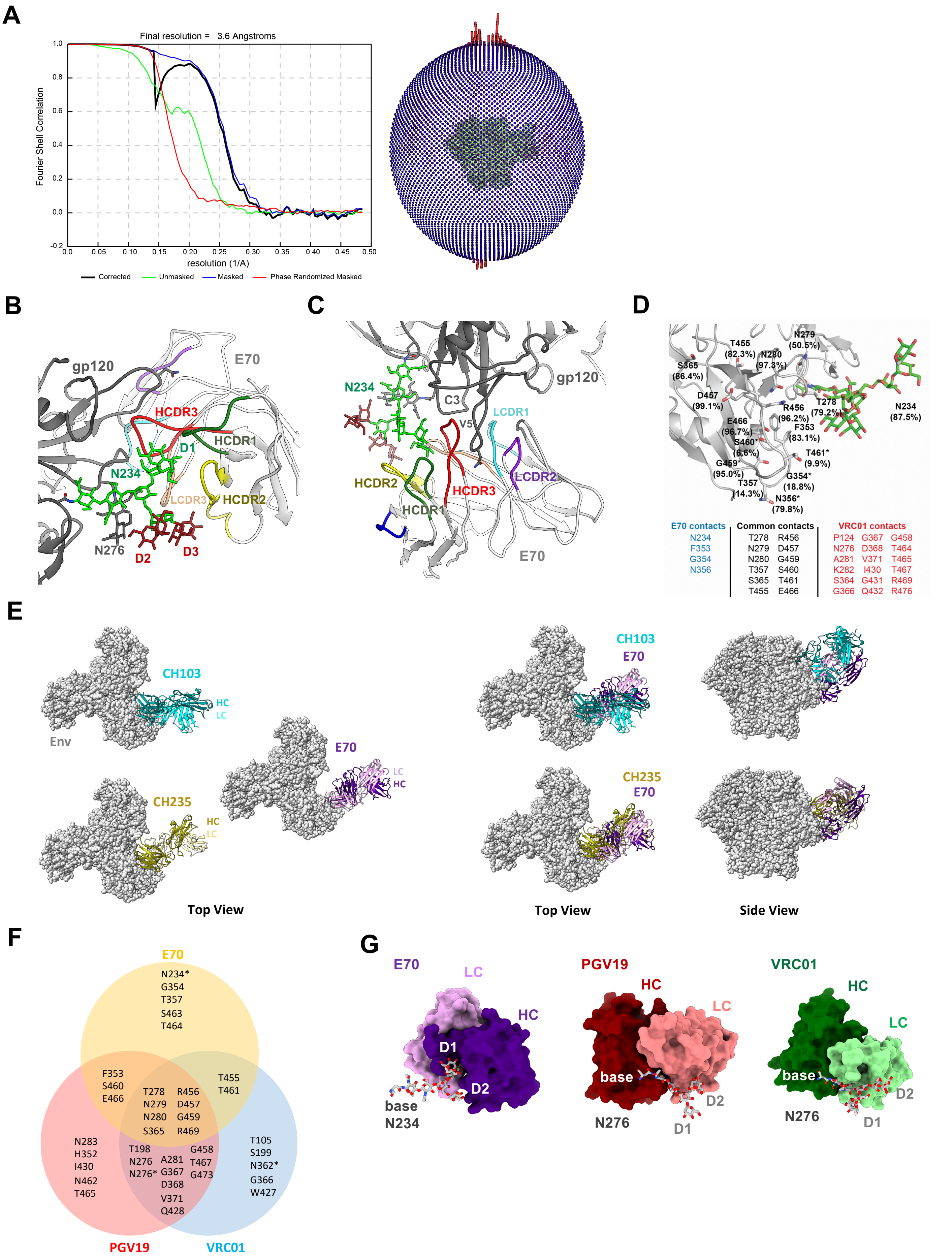
A
Final resolution = 3.6 Angstroms
Fourier Shell Correlation
resolution (1/A)
Corrected
Unmasked
Masked
Phase Randomized Masked
B
gp120
E70
HCDR3
HCDR1
D1
N234
LCDR3
HCDR2
N276
D2
D3
C
N234
C3
V5
gp120
LCDR1
LCDR2
HCDR2
HCDR3
HCDR1
E70
D
T455 (82.3%)
N279 (50.5%)
N280 (97.3%)
S365 (86.4%)
D457 (99.1%)
R456 (96.2%)
T278 (79.2%)
N234 (87.5%)
E466 (96.7%)
F353 (83.1%)
S460* (6.6%)
T461* (9.9%)
G459* (95.0%)
G354* (18.8%)
T357 (14.3%)
N356* (79.8%)
E70 contacts
N234 F353 G354 N356
Common contacts
T278 R456 N279 D457 N280 G459 T357 S460 S365 T461 T455 E466
VRC01 contacts
P124 G367 G458 N276 D368 T464 A281 V371 T465 K282 I430 T467 S364 G431 R469 G366 Q432 R476
E
CH103
HC
LC
Env
E70
LC
HC
CH235
HC
LC
Top View
CH103
E70
CH235
E70
Top View
Side View
F
E70
N234* G354 T357 S463 T464
F353 S460 E466
T455 T461
T278 R456 N279 D457 N280 G459 S365 R469
N283 H352 I430 N462 T465
T198 N276 N276* A281 G367 D368 V371 Q428 G458 T467 G473
T105 S199 N362* G366 W427
PGV19
VRC01
G
E70
LC
HC
D1
D2
base
N234
PGV19
HC
LC
base
N276
D2
D1
VRC01
HC
LC
base
N276
D2
D1

Figure S4. Epitope analysis reveals the CD4bs-directed E70 recognizes a chimeric N-glycan polypeptide epitope, related to Figure 4 and Table S5. (A) Fourier shell correlation (FSC) and orientation angle distribution of C3 symmetric reconstruction of BG505 NFL CC+ in complex with E70 Fab. (B) E70 Fab interactions with N234 glycan are primarily with the D1 arm. A complete Man9 glycoform is modeled (data supported sugars colored green; idealized model sugars colored dark red) to demonstrate that the D2 and D3 arms can be accommodated but do not contribute to the Ab epitope. Glycan N276 (grey) below is not involved in binding. (C) Additional E70 peptide contacts include C3 and V5 of gp120. (D) Amino acid contacts within the E70 epitope with conservation shown as a percentage. (E) Env binding angle comparison between E70 and non-VRC01 class Abs CH103 (PDB 4JAN) and CH235 (5F9W). (F) Overlap of contact residues between E70, VRC01, and PGV19. Asparagine residues marked with a star (*) denote that the contact is with a sugar molecule and not the amino acid side chain. Analysis based on a 4.0 Å distance cutoff between Fab and gp120 (VRC01 based on PDB 5FYK; PGV19 based on PDB 6B0N). Residues listed are of BG505 NFL TD CC+ (HXBc2 numbering). (G) Differences in glycan interactions between CD4bs-directed mAbs. VRC01 based on PDB 5FYK. PGV19 based on PDB 6B0N.

Figure S5

A

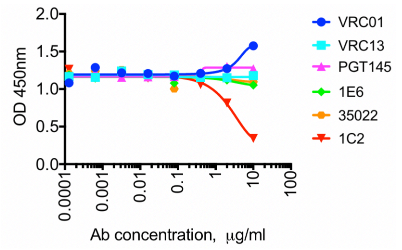

B

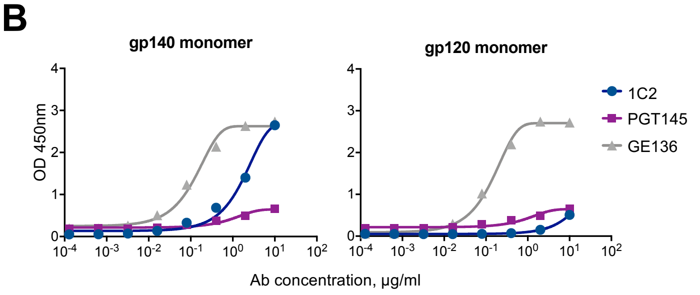

C

	JRFL						
	IC50 (µg/ml)				Fold Change		
	1 h	6 h	12 h	24 h	1h/6h	1h/12h	1h/24h
**1C2**	3.7	2.1	1.6	0.74	1.8	2.4	5.1
**3BC176**	0.25	0.14	0.086	0.026	1.9	2.9	9.5
**3BC315**	5.6	2.3	1.7	0.91	2.5	3.3	6.2
**PGV04**	0.0022	0.0028	0.0018	0.00090	0.8	1.2	2.4
**2G12**	0.33	0.28	0.25	0.20	1.2	1.3	1.6
**PGT128**	0.0021	0.0023	0.0015	0.00079	0.9	1.4	2.6
**PGT151**	0.0024	0.0016	0.0013	0.00065	1.5	1.9	3.7
**T-20**	0.015	0.013	0.013	0.014	1.2	1.2	1.1

	BG505						
	IC50 (µg/ml)				Fold Change		
	1 h	6 h	12 h	24 h	1h/6h	1h/12h	1h/24h
**1C2**	1.8	1.2	0.62	0.21	1.5	2.9	8.5
**3BC176**	2.0	1.2	0.7	0.35	1.7	2.7	5.8
**3BC315**	2.9	1.8	1.4	0.68	1.6	2.1	4.3
**PGV04**	0.0022	0.0019	0.0022	0.00098	1.2	1.0	2.3
**2G12**	>20	>20	>20	>20	NA	NA	NA
**PGT128**	0.068	0.048	0.070	0.031	1.4	1.0	2.2
**PGT151**	0.00013	0.00016	0.00014	0.00007	0.8	0.9	1.9
**T-20**	0.0043	0.0049	0.0048	0.0059	0.9	0.9	0.7

D

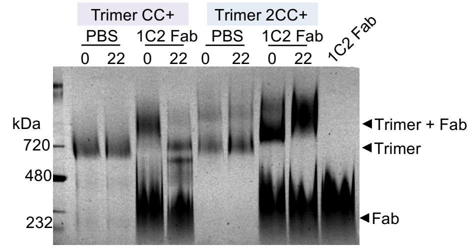

E

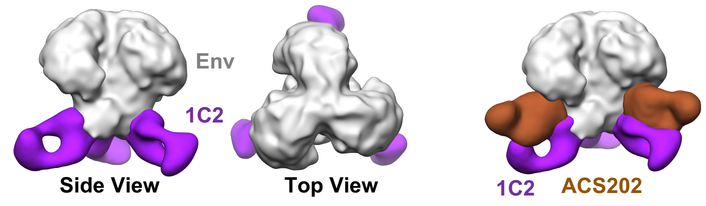

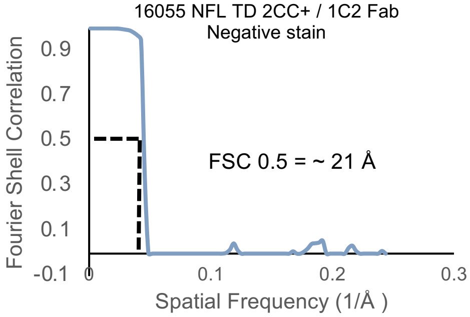

F

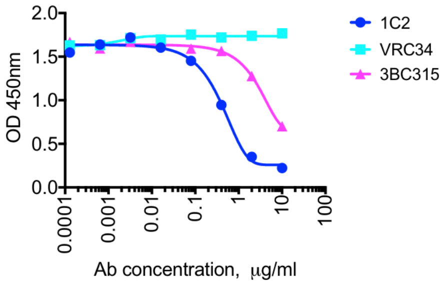

Figure S5. 1C2 binds the gp120:gp41 interface and destabilizes the Env trimer, related to Figure 5. (A) Cross-competition ELISA between rabbit mAb, 1C2, against a panel of human mAbs targeting different Env sites [CD4bs (VRC01, VRC13), V2-apex (PGT145), 35O22 (gp120:gp41)]. (B) Binding of 1C2 to 16055 gp140 monomer (left) compared to gp120 monomer (right) by ELISA. PGT145 (trimer-specific) and GE136 (monomer-specific) were used as negative and positive controls. (C) Antibodies noted on the left were pre-incubated with JRFL or BG505 virus for various times as indicated and then added to TZM-bl cells to test for infectivity. IC_{50} (µg/ml) values are noted with fold changes in IC_{50} values over time calculated relative to the standard 1-hr incubation time. (D) 1C2 Fab was pre-incubated for the indicated times with soluble 16055 NFL TD CC+ or 2CC+ trimers prior to separation by blue-native PAGE. (E) Left, EM 3D reconstruction model of 1C2 Fab (purple) bound to 16055 NFL TD 2CC+ trimer with the Fourier shell correlation graph shown below. Right, another gp120:gp41-directed bNAb, ACS202 (EMD-8299) is docked into the 1C2:16055 NFL TD 2CC+ model for comparison. (F) Cross-competition ELISA between 1C2 and VRC34 (FP-directed) and 3BC315 (gp120:gp41).

Figure S6

A

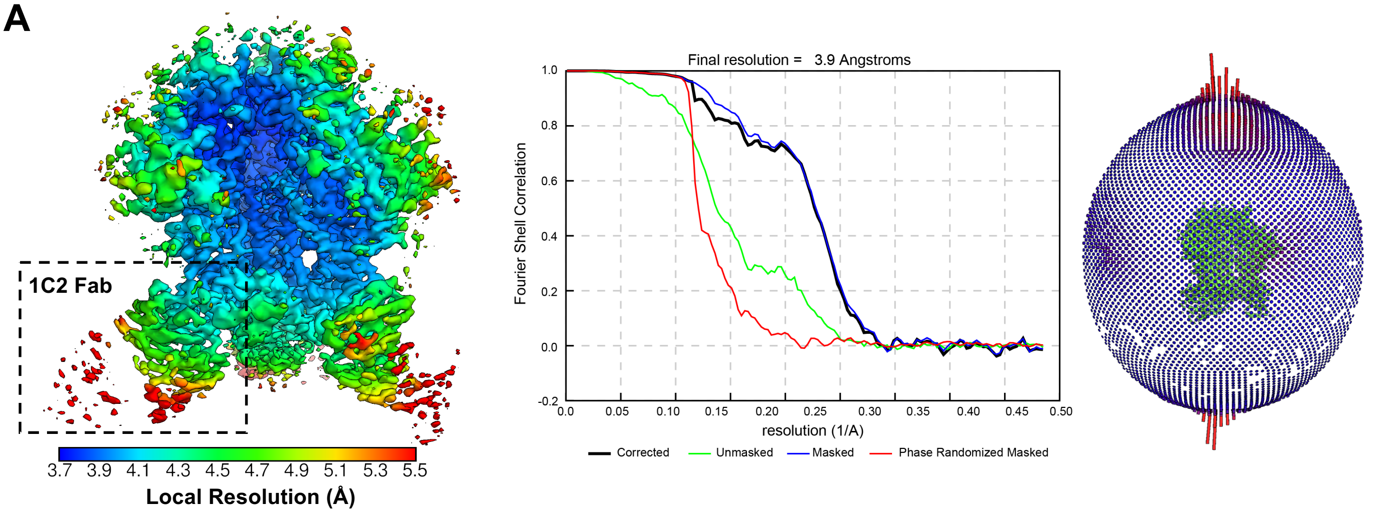

B

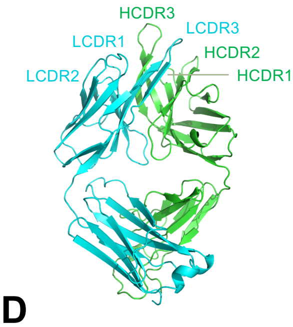

C

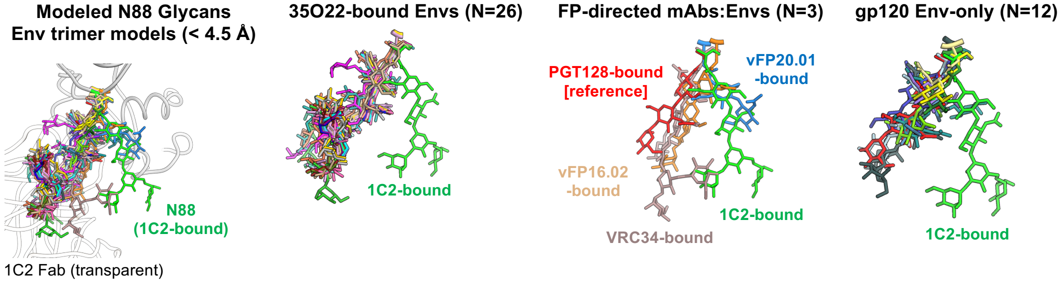

D

	1C2	3BC315	ACS202	VRC01
JRFL WT	1.12	33.17	0.04	0.02
N88A	0.29	0.23	>50	0.03
N611A	6.66	14.10	0.01	0.03
N625A	1.65	11.29	0.02	0.02

E

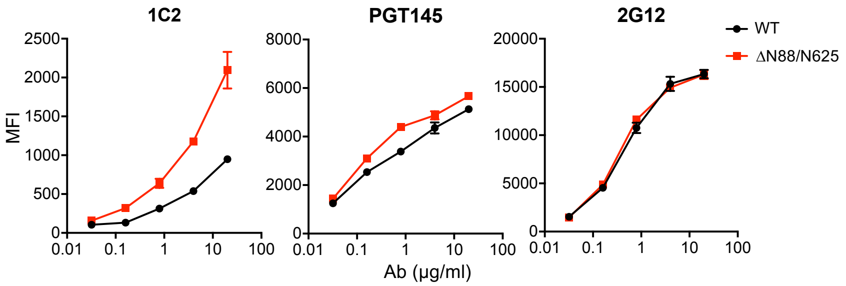

F

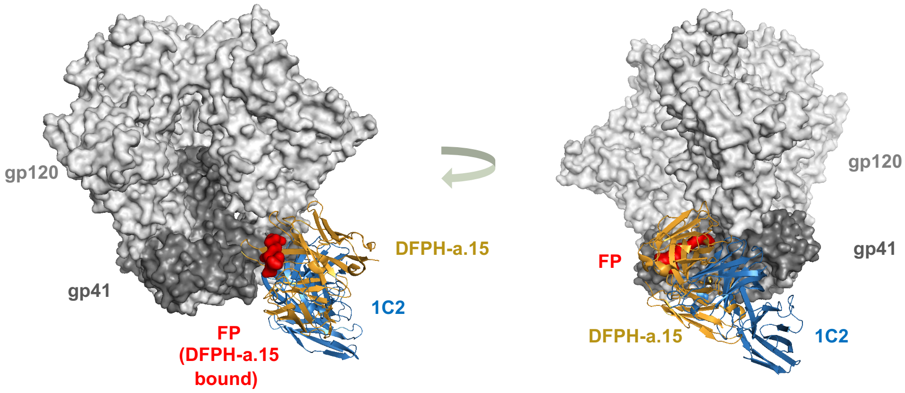

Figure S6. Epitope analysis and high resolution cryoEM structure of 1C2 bound-Env trimer reveals complex interface recognition, related to Figure 6 and Tables S5-S6. (A) ~3.9 Å resolution reconstruction of 16055 NFL TD 2CC+ trimer in complex with 1C2 Fab, colored by local resolution according to the key. One Fab is boxed for reference. To the right, the Fourier shell correlation (FSC) and orientation angle distribution of C3 symmetric reconstruction of 16055 NFL TD 2CC+ in complex with 1C2 Fab are shown. (B) Crystal structure of unliganded 1C2 Fab. Heavy (green) and light (cyan) chains are shown in ribbon representation. The CDRs are highlighted. (C) Comparison of the N88 glycan conformation between the 1C2-bound 16055 NFL TD 2CC+ structure (green) and other published HIV-1 trimer structures with near atomic resolution (< 4.5 Å) that have modeled N88 glycans (n = 42). Subset comparisons between 35O22-bound Env trimers, FP-directed mAbs (vFP20.01, PDB 6CDE; vFP16.02, PDB 6CDI; VRC34, PDB 5I8H) with the N88 from the PGT128-bound Env (PDB 5ACO) shown as a reference, and gp120 Env-only structures are presented. (D) Neutralization sensitivity of gp120:gp41-directed mAbs against a panel of JRFL N-glycan mutants around the interface. VRC01 (CD4bs-directed) is included as a negative control. IC50 (μg/ml) values are shown. (E) 1C2 binding to cell surface expressed WT BG505 Env compared to that with the N88 and N625 PNGS genetically removed (ΔN88/N625), as assessed by FACs. PGT145 and 2G12 binding were included as trimer expression controls. (F) Comparison of Env (gp120, light grey; gp41 dark grey) recognition by 1C2 (blue) and the vaccine-elicited FP targeting mAb DFPH-a.15 (orange; PDB 6N1W). Interaction with a single protomer is shown for clarity with the FP highlighted in red.

Figure S7

Virus ID	Clade	1C2	3BC315
0260.v5.c36	A	7.8500	>200
0330.v4.c3	A	1.4200	3.9200
0439.v5.c1	A	18.4000	>200
3365.v2.c20	A	17.6000	>200
3415.v1.c1	A	41.8000	>200
3718.v3.c11	A	35.1000	24.8000
398-F1.F6.20	A	7.7200	7.2100
BB201.B42	A	4.7400	6.8500
BB539.2B13	A	3.5100	1.3300
BG505.W6M.C2	A	8.9300	8.9200
BI369.9A	A	7.5700	12.6000
BS208.B1	A	0.2370	0.3100
KER2008.12	A	5.6900	2.5000
KER2018.11	A	16.8000	20.8000
KNH1209.18	A	14.0000	>200
MB201.A1	A	3.3400	7.8500
MB539.2B7	A	26.6000	3.5800
MI369.A5	A	14.2000	33.7000
MS208.A1	A	0.7190	0.8710
Q23.17	A	10.7000	14.7000
Q259.17	A	15.3000	5.7000
Q769.d22	A	2.2000	0.7100
Q769.h5	A	15.0000	2.8000
Q842.d12	A	10.8000	28.2000
QH209.14M.A2	A	3.6700	3.6900
RW020.2	A	17.1000	>200
UG037.8	A	25.8000	80.8000
246-F3.C10.2	AC	2.4500	0.5400
3301.v1.c24	AC	9.1000	80.1000
3589.v1.c4	AC	11.0000	>200
6540.v4.c1	AC	27.6000	15.9000
6545.v4.c1	AC	10.9000	10.0000
0815.v3.c3	ACD	11.3000	6.9600
6095.v1.c10	ACD	0.3170	>200
3468.v1.c12	AD	37.9000	>200
Q168.a2	AD	14.5000	8.2300
Q461.e2	AD	>200	>200
620345.c1	AE	31.5000	>200
BJOX009000.02.4	AE	16.4000	14.3000
BJOX010000.06.2	AE	10.6000	37.2000
BJOX025000.01.1	AE	6.1600	78.3000
BJOX028000.10.3	AE	7.3500	>200
C1080.c3	AE	3.7700	28.2000
C2101.c1	AE	5.8600	>200
C3347.c11	AE	>200	>200
C4118.09	AE	9.1400	>200
CM244.ec1	AE	26.3000	15.5000
CNE3	AE	30.4000	63.8000
CNE5	AE	46.9000	>200
CNE55	AE	7.3400	4.1400
CNE56	AE	30.9000	3.5600
CNE59	AE	0.0840	0.0940

Virus ID	Clade	1C2	3BC315
CNE8	AE	12.4000	6.7400
M02138	AE	0.4570	0.8490
R1166.c1	AE	7.3800	7.6700
R2184.c4	AE	7.0600	>200
R3265.c6	AE	9.1200	>200
TH023.6	AE	0.0020	0.0120
TH966.8	AE	24.4000	4.7200
TH976.17	AE	6.1700	>200
235-47	AG	15.1000	57.7000
242-14	AG	6.1100	>200
263-8	AG	4.4500	0.4170
269-12	AG	0.9310	>200
271-11	AG	33.6000	>200
928-28	AG	12.4000	>200
DJ263.8	AG	1.8700	######
T250-4	AG	11.3000	>200
T251-18	AG	6.0300	>200
T253-11	AG	40.8000	>200
T255-34	AG	3.0100	>200
T257-31	AG	>200	16.4000
T266-60	AG	>200	>200
T278-50	AG	2.5000	>200
T280-5	AG	18.4000	16.4000
T33-7	AG	19.6000	>200
3988.25	B	3.5700	4.8200
5768.04	B	34.5000	30.8000
6101.10	B	0.6710	1.0300
6535.3	B	9.7800	>200
7165.18	B	9.5500	10.4000
45_01dG5	B	11.9000	18.1000
89.6.DG	B	2.6700	>200
AC10.29	B	4.1600	7.1800
ADA.DG	B	28.8000	>200
Bal.01	B	14.2000	12.7000
BaL.26	B	19.0000	13.7000
BG1168.01	B	9.5500	11.3000
BL01.DG	B	20.1000	14.0000
BR07.DG	B	5.7300	3.6200
BX08.16	B	5.7900	2.8400
CAAN.A2	B	6.5400	>200
CNE10	B	7.0000	3.9200
CNE12	B	10.3000	9.6400
CNE14	B	30.6000	######
CNE4	B	5.8100	>200
CNE57	B	7.8500	6.8200
HO86.8	B	4.0400	11.1000
HT593.1	B	2.2100	0.9390
HXB2.DG	B	16.4000	>200
JRCSF.JB	B	34.5000	50.5000
JRFL.JB	B	10.8000	19.0000
MN.3	B	0.2950	0.3860
PVO.04	B	25.2000	>200

Virus ID	Clade	1C2	3BC315
QH0515.01	B	61.1000	38.8000
QH0692.42	B	11.9000	32.1000
REJO.67	B	6.8800	9.1800
RHPA.7	B	4.7500	2.3800
SC422.8	B	13.2000	19.5000
SF162.LS	B	6.9400	11.3000
SS1196.01	B	51.7000	>200
THRO.18	B	6.8400	######
TRJO.58	B	5.1700	23.0000
TRO.11	B	5.4700	3.4300
WITO.33	B	5.5600	>200
X2278.C2.B6	B	>200	>200
YU2.DG	B	7.6300	5.2900
BJOX002000.03.2	BC	22.0000	32.9000
CH038.12	BC	>200	>200
CH070.1	BC	>200	######
CH117.4	BC	4.0200	>200
CH119.10	BC	2.5700	>200
CH181.12	BC	51.2000	61.1000
CNE15	BC	21.5000	9.1700
CNE19	BC	2.1000	>200
CNE20	BC	1.5200	>200
CNE21	BC	6.1000	>200
CNE40	BC	0.3900	######
CNE7	BC	12.0000	>200
286.36	C	24.0000	5.3200
288.38	C	7.2500	1.8400
0013095-2.11	C	8.9500	11.7000
001428-2.42	C	4.4200	2.5600
0077.v1.c16	C	14.5000	######
00836-2.5	C	13.4000	6.2800
0921.v2.c14	C	27.2000	21.5000
16055-2.3	C	5.8500	42.9000
16845-2.22	C	>200	>200
16936-2.21	C	4.6400	>200
25710-2.43	C	1.5900	0.7520
25711-2.4	C	12.8000	>200
25925-2.22	C	4.9000	22.8000
26191-2.48	C	42.6000	94.5000
3168.v4.c10	C	4.9000	>200
3637.v5.c3	C	62.9000	92.3000
3873.v1.c24	C	>200	99.4000
426c	C	11.9000	7.8200
6322.v4.c1	C	6.6300	4.9100
6471.v1.c16	C	>200	>200
6631.v3.c10	C	######	>200
6644.v2.c33	C	0.0630	0.0690
6785.v5.c14	C	29.5000	>200
6838.v1.c35	C	7.6700	>200
96ZM651.02	C	1.7400	9.2000
BR025.9	C	4.3600	14.1000
CAP210.E8	C	8.2000	99.8000

Virus ID	Clade	1C2	3BC315
CAP244.D3	C	0.5870	>200
CAP256.206.C9	C	>200	>200
CAP45.G3	C	25.1000	47.4000
Ce1176.A3	C	5.9700	######
CE703010217.B6	C	9.7000	>200
CNE30	C	10.7000	######
CNE31	C	18.2000	>200
CNE53	C	10.6000	2.1700
CNE58	C	7.1800	82.3000
DU123.06	C	2.6600	######
DU151.02	C	3.4500	2.7500
DU156.12	C	>200	>200
DU172.17	C	>200	>200
DU422.01	C	4.3800	6.8900
MW965.26	C	0.1710	>200
SO18.18	C	20.8000	>200
TV1.29	C	######	39.7000
TZA125.17	C	>200	>200
TZBD.02	C	>200	>200
ZA012.29	C	6.2300	>200
ZM106.9	C	6.9800	5.8400
ZM109.4	C	4.5800	1.1900
ZM135.10a	C	5.7100	94.0000
ZM176.66	C	12.7000	43.6000
ZM197.7	C	3.1400	3.1100
ZM214.15	C	10.5000	33.8000
ZM215.8	C	2.8300	5.2700
ZM233.6	C	2.8500	>200
ZM249.1	C	7.7100	14.2000
ZM53.12	C	>200	>200
ZM55.28a	C	28.9000	6.5700
3326.v4.c3	CD	38.0000	>200
3337.v2.c6	CD	7.2200	>200
3817.v2.c59	CD	65.1000	>200
191821.E6.1	D	27.8000	62.7000
231965.c01	D	57.0000	>200
247-23	D	51.5000	7.5600
3016.v5.c45	D	######	7.0900
57128.vrc15	D	8.2400	>200
6405.v4.c34	D	4.5400	6.4200
A03349M1.vrc4a	D	6.0200	>200
A07412M1.vrc12	D	11.2000	74.1000
NKU3006.ec1	D	11.9000	>200
UG021.16	D	6.9400	2.8900
UG024.2	D	5.2100	2.9900
P0402.c2.11	G	33.3000	>200
P1981.C5.3	G	0.8600	>200
X1193.c1	G	39.1000	>200
X1254.c3	G	######	>200
X1632.S2.B10	G	32.3000	>200
X2088.c9	G	8.9300	57.8000
X2131.C1.B5	G	5.8900	>200
SIVmac251.30.SG3	NA	>200	>200

	1C2		3BC315	
# Viruses Neutralized	#	%	#	%
IC50 <200ug/ml	192	92	127	61
IC50 <50ug/ml	181	87	102	49
IC50 <10ug/ml	105	50	63	30
IC50 <1.0ug/ml	14	7	12	6
IC50 <0.1ug/ml	3	1	3	1
IC50 <0.01ug/ml	1	0	0	0
Total # Viruses	208			

	1C2	3BC315
Median IC50	8.93	10.00
Geometric Mean	8.03	10.05

*Median and Geometric Mean titers calculated only for samples with IC50 <50ug/ml

Figure S7. 1C2 neutralization breadth is broader than the human bNAb 3BC315, related to Figure 6. Neutralization IC_{50} (µg/ml) values shown for 1C2 and 3BC315 against a diverse 208-virus panel.

Table S1. Site-specific glycan analysis of N-glycan deleted trimers compared to WT, related to Figure 1. Percentage point differences in glycan abundances for glycan-deleted constructs compared to WT. A decrease in glycan abundance greater than 10 percentage points is highlighted in increasing shades of red, whereas an increase in abundance is highlighted in green. Knocked-out glycans are highlighted in blue.

16055

ΔGly1	88	160	187D	197	262	276	301	332	360	386	442	448	463	625	637
M9	-0.3	0.7	-0.2	-5.1	**-13.2**	K/O	-6.1	-9.6	**-13.4**	**-15.4**	**-14.4**	**-11.4**	-0.2	-0.4	**-15.1**
M8	-0.6	5.7	-0.7	1.2	4.0		-2.6	6.9	4.5	**11.7**	5.1	1.1	-0.3	-0.4	-0.3
M7	-0.7	-3.9	-0.7	0.4	2.5		-6.7	2.2	1.2	6.5	4.1	1.9	0.0	-0.5	-3.2
M6	-3.1	3.6	-0.4	0.2	3.1		3.5	-0.1	1.1	1.3	1.7	0.9	-0.1	-0.4	-0.9
M5	3.4	-5.3	-6.1	-1.7	3.7		-0.1	-0.2	4.0	0.2	2.6	-0.6	-0.3	-0.4	0.2

ΔGly2	88	160	187D	197	262	276	301	332	360	386	442	448	463	625	637
M9	0.0	-0.6	-0.1	-4.9	**-16.2**	K/O	-2.9	**-15.3**	**-40.1**	**-17.4**	**-20.1**	**-17.2**	K/O	-0.1	**-16.5**
M8	0.6	1.8	0.2	0.7	7.1		**-16.6**	6.8	9.0	**11.0**	2.9	-7.2		0.5	-0.4
M7	-0.1	-2.4	0.0	0.5	3.7		5.3	3.2	6.2	5.9	3.6	4.5		0.4	-2.4
M6	-2.7	2.7	0.2	0.0	0.0		3.9	0.4	3.2	0.0	1.5	0.7		0.0	-1.0
M5	0.7	-1.3	-2.8	0.6	5.4		8.5	0.1	**10.8**	0.0	4.5	0.0		-1.4	2.7

ΔGly4	88	160	187D	197	262	276	301	332	360	386	442	448	463	625	637
M9	0.2	**-13.0**	-0.2	-6.8	-8.0	K/O	K/O	**-29.9**	K/O	**-23.8**	**-42.5**	**-23.2**	K/O	0.5	N.D.
M8	0.8	-1.5	1.3	-2.0	1.5			9.7		9.3	**-19.0**	0.7		1.1	N.D.
M7	1.3	1.7	1.0	1.2	1.7			5.3		6.0	9.9	8.0		1.5	N.D.
M6	-0.5	4.7	0.3	0.3	1.5			1.0		0.0	**25.9**	2.7		0.5	N.D.
M5	-5.5	7.4	-6.8	2.1	3.2			0.3		0.0	**16.7**	1.1		4.3	N.D.

JRFL

ΔGly1	88	156	160	241	262	276	295	332	339	356	362	386	448	463	616	625	637
M9	0.0	4.1	-1.5	-6.5	**-24.3**	K/O	-9.0	**-11.8**	-3.4	-0.1	**-38.7**	-5.7	**-18.2**	-0.4	-0.1	-0.1	0.8
M8	0.1	2.6	1.2	0.3	**15.3**		9.0	1.0	**-13.6**	0.0	**27.3**	7.3	4.2	-0.7	-0.1	0.1	**-10.4**
M7	0.7	-2.7	-0.2	-2.9	2.4		0.0	-0.2	-8.9	0.3	6.5	0.0	2.0	-0.4	0.0	0.4	**-17.8**
M6	1.5	-1.8	0.3	-0.9	0.0		0.0	-0.1	-0.7	0.1	1.6	0.0	1.0	0.2	0.0	0.6	-8.7
M5	4.0	-1.7	1.0	2.9	6.5		0.0	3.8	1.7	0.8	2.1	0.0	1.5	3.1	1.6	4.7	-0.4

ΔGly2	88	156	160	241	262	276	295	332	339	356	362	386	448	463	616	625	637
M9	0.0	4.2	-4.8	-4.7	**-28.0**	K/O	-4.0	**-15.3**	-3.7	-0.1	**-42.3**	-7.0	**-27.4**	K/O	-0.1	-0.1	0.0
M8	0.0	1.1	-3.7	-4.6	**21.1**		4.0	-6.0	**-17.1**	0.0	**12.8**	5.3	-1.2		0.1	0.3	**-11.6**
M7	0.3	-2.8	0.4	-8.9	2.9		0.0	0.3	**-12.7**	0.3	**10.5**	0.0	3.7		0.3	0.7	**-20.1**
M6	-1.0	-1.6	0.5	-2.2	0.8		0.0	-0.4	-4.3	0.2	8.1	0.0	1.6		0.2	0.7	-9.9
M5	2.8	-0.3	3.1	5.7	1.2		0.0	5.6	-1.0	1.4	5.3	0.0	2.1		3.0	2.8	1.5

BG505

ΔGly1	88	160	190	197	234	262	276	295	332	339	355	363	386	448	462	611	618	637
M9	0.0	**-29.0**	-0.1	**-14.9**	**-55.5**	**-36.9**	K/O	**-21.4**	**-33.8**	**-23.7**	-0.2	**-28.1**	**-24.7**	**-38.4**	0.0	0.0	0.0	-1.1
M8	-0.4	**14.7**	-0.7	2.7	**27.0**	**17.9**		**14.8**	**11.7**	7.0	-0.4	**13.9**	**17.7**	6.2	-0.3	-1.5	0.0	**-15.7**
M7	-0.3	7.4	-0.3	4.1	**12.9**	5.2		4.1	**10.7**	4.0	-0.9	6.3	4.8	**10.3**	-0.3	0.0	0.0	**-32.1**
M6	-1.1	4.3	-0.1	6.1	**10.4**	**13.3**		2.5	2.2	1.2	-0.6	4.4	0.0	7.2	-0.1	0.3	0.0	**-14.4**
M5	-1.7	2.6	-1.2	-0.7	5.0	0.5		0.0	-0.1	0.3	**-14.1**	2.5	0.0	4.8	-0.1	3.4	1.0	**20.1**

ΔGly2	88	160	190	197	234	262	276	295	332	339	355	363	386	448	462	611	618	637
M9	0.0	**-35.4**	-0.1	**-21.0**	**-69.1**	**-44.0**	K/O	**-36.1**	**-38.7**	**-32.5**	-0.2	**-37.1**	**-52.6**	**-45.3**	K/O	0.0	0.0	-1.1
M8	-0.2	**15.9**	-0.3	-3.0	**35.0**	**20.9**		**13.6**	6.8	4.7	-0.3	**19.5**	**41.7**	-1.9		-0.4	0.0	**-15.7**
M7	-0.2	7.9	-0.6	3.0	**16.7**	8.6		5.0	6.0	4.8	-1.0	3.2	9.7	7.9		0.7	0.0	**-29.5**
M6	-0.7	4.8	0.1	5.3	**12.1**	**13.4**		3.1	1.9	1.8	-0.8	7.9	0.0	8.6		1.8	0.0	**-14.4**
M5	-2.3	4.7	-0.6	2.2	2.6	1.1		0.0	-0.1	0.4	**-19.8**	4.0	0.0	8.8		**12.5**	-0.1	**14.9**

Table S2. mAbs cloned from rabbit C3, related to Figures 3, 5, S3, and Table S3 and S4.
Env binding (+), # of mAbs with positive binding to Env by ELISA. Neutralizing, # of mAbs capable of neutralizing ≥1 HIV-1 strains.

Set#	Probes	#Cells Sorted	#mAbs Cloned	Productive	Env Binding (+)	Neutralizing
1	16066 gp120+<br>16055 gp120 368R/474A-	176	15	13	13	5
2	16055 gp120+<br>16055 gp120 368R-	88	12	2	2	0
3	16066 gp120+<br>SC422 NFL TD CC+	88	2	1	1	1
4	16055 NFL TD CC+<br>JRFL NFL TD14	121	3	2	2	2
5	16055 NFL TD CC+<br>16055 gp120 ΔGly4	14	10	1	0	0
6	16055 NFL TD CC+	118	10	7	4	4
7	16055 gp120<br>16055 gp120 ΔGly4	70	11	4	2	1
8	16055 NFL TD CC+<br>1086 NFL TD CC+	116	36	20	19	8
	**Total**	**791**	**99**	**50**	**43**	**21**

Table S3. Rabbit single-cell PCR primers, related to Figures 3, 5, S3 and Tables S2 and S4. Supplemental primers to those reported in McCoy et al., 2016.

PCR step	Primer Name	5'-3' sequence
Kappa PCR 1	KFOut1	GRACAYGAGGGCCCCCACTCAG
Kappa PCR 1	KFOut2	CYAGSCAGGACCCAGCATG
Kappa PCR 1	KROut1	CATCCACCTCCCAGGTGACGG
Kappa PCR 1	KROut2	CATCCACCTTCCAGGTGACGGT
Kappa PCR 1	KROut3	CATCCACCTGCCAGGTGACGG
Kappa PCR 2	KFIn1	GMTGGGGCTCCTRCTGC
Kappa PCR 2	KRIn2	CACACGATGGTGACTGTTTCAGTTG
Kappa PCR 2	KRIn3	CACACGATGGTGGCTGTTCC
Heavy PCR 1	HForOut	GGCTTCTCCTGGTCGCTGTGCTCAAAGGTGTC
Heavy PCR 1	HROut1	GATCATGCAGGTCAGGCTGACCGACCTGC
Heavy PCR 1	HROut2	GGCTTCTGCAGAGCTCCCGACTCCTGTC
Heavy PCR 1	RH1OutR	GACACACCCAGCTCCACGGTGAC
Heavy PCR 2	RH1inR	GCCAGTGGGAAGACTGAYGGAG
Heavy PCR 2	HVForIn	GCTGTGCTCAAAGGTGTCCAGTGTC
Heavy PCR 2	HVRevIn	CAGTGGGAAGACTGAYGGAGCCTTAG

Table S4. Profile of neutralizing mAbs cloned from rabbit C3, related to Figures 3, 5, S3, and Tables S2-S3. Tissue refers to the tissue sample from which the B cell were sorted; LN, lymph nodes, PBMCs, peripheral blood mononuclear cells. (+) indicates positive binding to Env by ELISA. Neutralization IC_{50} (μg/ml) values are indicated and colored by potency.

#	mAb	Tissue	Probes	Env Binding	Tier 1 Viruses MN.3	Tier 1 Viruses HXBc2	Tier 2 Viruses 16055	Tier 2 Viruses Ce1176	Tier 2 Viruses TRO.11
1	A44	Spleen	16055 gp120+<br>16055 gp120 368R/474A-	+	>50	>50	19.11	>50	>50
2	A147			+	0.001	>50	>50	>50	>50
3	A163			+	0.05	>50	>50	>50	>50
4	A170			+	0.01	>50	>50	>50	>50
5	A174			+	>50	0.41	>50	>50	>50
6	E70	Spleen	16055 gp120/SC422 NFL TD CC+	+	>50	>50	0.454	0.15	3.551
7	F5	Spleen	16055 NFL TD CC+<br>JRFL NFL TD14	+	>50	>50	5.57	>50	>50
8	G70	Spleen		+	0.018	0.08	>50	>50	>50
9	2D5	LN		+	0.48	>50	26.82	>50	>50
10	B5	LN	16055 NFL TD CC+	+	1.88	>50	>50	>50	>50
11	D4			+	0.13	>50	>50	>50	>50
12	F4			+	0.09	>50	>50	>50	>50
13	H2			+	0.5	>50	>50	>50	>50
14	1C2	LN	16055 NFL TD CC+	+	0.3	17.04	4.56	4.46	21.38
15	1D1			+	1.8	>50	>50	>50	>50
16	1D6			+	13.02	6.31	>50	>50	>50
17	1G9			+	0.61	0.22	>50	>50	>50
18	1F4		1086 NFL TD CC+	+	6.09	>50	>50	>50	>50
19	2A6			+	0.18	>50	>50	>50	>50
20	2C5			+	0.02	>50	21.38	>50	>50
21	A10	PBMcs	16055 gp120/16055 gp120 ΔGly4	+	1.23	0.76	>50	>50	>50

Table S5. CryoEM data collection and processing statistics, related to Figures 4, 6, S4, and S6.

**Map**	**E70 Fab:BG505 NFL CC+**	**1C2 Fab:16055 NFL TD 2CC+**
**Data collection**		
Microscope	FEI Titan Krios	FEI Talos Arctica
Voltage (kV)	300	200
Detector	Gatan K2 Summit	Gatan K2 Summit
Recording mode	Counting	Counting
Nominal magnification	29,000	36,000
Movie micrograph pixelsize (Å)	1.03	1.15
Dose rate (e^-/[(camera pixel)*s])	5.04	5.10
Number of frames per movie micrograph	48	50
Frame exposure time (ms)	250	250
Movie micrograph exposure time (s)	12.0	12.5
Total dose ($e^-/Å^2$)	57	48
Defocus range (μm)	-0.5 to -2.0	-0.7 to -2.5
**EM data processing**		
Number of movie micrographs	870	1,690
Number of molecular projection images in map	49,635	23,702
Symmetry	C3	C3
Map resolution (FSC 0.143; Å)	3.57	3.94
Local resolution range (Å)[1]	3.4-5.2	3.7-5.5
Map sharpening B-factor ($Å^2$)	-138	-111
**Structure Building and Validation**		
Number of atoms in deposited model		
NFL Env	13,029	13,209
Fab Fv	5,061	5,295
glycans	1,113	1,815
MolProbity score	1.13	0.95
Clashscore	1.09	1.75
Map correlation coefficient	0.82	0.81
EMRinger score	3.69	2.77
RMSD from ideal		
Bond length (Å)	0.02	0.02
Bond angles (˚)	1.83	1.84
Ramachandran plot		
Favored (%)	95.41	97.94
Allowed (%)	4.59	2.06
Outliers (%)	0.00	0.00
Side chain rotamer outliers (%)	0.15	0.44

[1]In modeled regions of map; based on Relion 3.0 Local Resolution function

Table S6. Crystallization data collection and refinement statistics for 1C2 Fab, related to Figures 6 and S6.

	1C2 unliganded
**Data collection**	
Space group	$P22_12_1$
Cell dimensions	
a, *b*, *c* (Å)	71.80, 98.60, 162.98
α, *β*, *γ* (°)	90, 90, 90
Resolution (Å)	50-2.30 (2.34-2.30)*
R_{sym} or R_{merge}	0.071 (0.723) *
I/s*I*	12.4 (1.4)*
Completeness (%)	88.0 (80.4)*
Redundancy	3.7 (2.8)*
$CC_{1/2}$	0.998 (0.686)*
**Refinement**	
Resolution (Å)	35.44-2.296 (2.378-2.296)*
No. reflections	42,972
R_{work}/R_{free}	21.92/25.42 (29.74/33.57)*
No. atoms	6753
Protein	6475
Water	268
Ligand	10
B-factors ($Å^2$)	41.64
Protein	41.71
Water	38.54
Ligand	78.05
R.m.s deviations	
Bond lengths (Å)	0.004
Bond angles (º)	0.72
Ramachadran Favored %	95.35
Ramachadran Outliers %	0.23
MolProbity all-atoms clashscore	1.96
